# Phenotypic and genetic characterization of a near-isogenic line pair: insights into flowering time in chickpea

**DOI:** 10.1186/s12870-024-05411-y

**Published:** 2024-07-25

**Authors:** Adrian Perez-Rial, Alejandro Carmona, Latifah Ali, Josefa Rubio, Teresa Millan, Patricia Castro, Jose V. Die

**Affiliations:** 1 Department of Genetics-ETSIAM, University of Córdoba, Campus de Rabanales, Córdoba, 14071 Spain; 2 Department of Plant Biology-Science Faculty, University of Tishreen, Lattakia City, Syria; 3 Área de Mejora y Biotecnología, IFAPA Centro ‘Alameda del Obispo’, Córdoba, 14080 Spain

**Keywords:** *Cicer arietinum*, Early-flowering, NILs, Sequencing, SNPs

## Abstract

**Background:**

*Cicer arietinum* is a significant legume crop cultivated mainly in short-season environments, where early-flowering is a desirable trait to overcome terminal constraints. Despite its agricultural significance, the genetic control of flowering time in chickpea is not fully understood. In this study, we developed, phenotyped, re-sequenced and genetically characterized a pair of near-isogenic lines (NILs) with contrasting days to flowering to identify candidate gene variants potentially associated with flowering time.

**Results:**

In addition to days to flowering, noticeable differences in multiple shoot architecture traits were observed between the NILs. The resequencing data confirms that the NILs developed in this study serve as appropriate plant materials, effectively constraining genetic variation to specific regions and thereby establishing a valuable resource for future genetic and functional investigations in chickpea research. Leveraging bioinformatics tools and public genomic datasets, we identified homologs of flowering-related genes from *Arabidopsis thaliana*, including *ELF3* and, for the first time in chickpea, *MED16* and *STO/BBX24*, with variants among the NILs. Analysis of the allelic distribution of these genes revealed their preservation within chickpea diversity and their potential association with flowering time. Variants were also identified in members of the ERF and ARF gene families. Furthermore, in silico expression analysis was conducted elucidating their putative roles in flowering.

**Conclusions:**

While the gene *CaELF3a* is identified as a prominent candidate, this study also exposes new targets in chickpea, such as *CaMED16b* and LOC101499101 (*BBX24-like*), homologs of flowering-related genes in *Arabidopsis*, as well as *ERF12* and *ARF2*. The in silico expression characterization and genetic variability analysis performed could contribute to their use as specific markers for chickpea breeding programs. This study lays the groundwork for future investigations utilizing this plant material, promising further insights into the complex mechanisms governing flowering time in chickpea.

**Supplementary Information:**

The online version contains supplementary material available at 10.1186/s12870-024-05411-y.

## Background

The domesticated chickpea (*Cicer arietinum* L.) is an annual and self-pollinated legume belonging to the Papilionoideae subfamily. Its genome size is estimated to be approximately 738 Mb (2*n* = 2*x* = 16), with the reference genome of the CDC Frontier kabuli cultivar assembled into 530 Mb [1]. Chickpea is the second most cultivated grain legume globally, with a production of 18.1 million tons across 14.8 million ha, yielding 1.22 t/ha in 2022 [2]. Despite its importance, global chickpea cultivation predominantly occurs in short-season environments, which expose the crop to terminal stresses, consequently limiting its potential yield [3–5]. In Mediterranean and semi-arid environments, terminal drought and heat are the primary causes of yield loss [6–9]. Conversely, in higher latitude areas like Canada, the growing season is affected by lower temperatures, delayed maturation, and an increased risk of frost damage [10–13]. In response to these challenges, early-flowering is a desirable trait for chickpea, acting as an effective escape strategy in various environmental conditions [5, 8, 10, 11, 14]. The flowering time is crucial to plant decisions about resource allocation and is involved in a complex web of interactions with other developmental processes. While the transition from vegetative to the reproductive phase is marked by the conversion of meristems to produce flowers instead of vegetative buds, it is also accompanied by significant changes in a range of other developmental traits, such as stem elongation and lateral branching. Despite growing interest, the genetic control of this complex trait remains unclear [15–17].

Chickpea, along with other notable legumes such as pea, lentil, and faba bean, belongs to the galegoid clade. Members of this clade originate from temperate regions and exhibit long-day flowering characteristics. In contrast, legumes in the phaseoloid clade, including soybean, cowpea, pigeon pea, and common bean, primarily come from lower latitudes and are identified as short-day plants [15]. Much of our current understanding of flowering time regulation stems from studies on the model long-day species *Arabidopsis thaliana* (L.) Heynh*.*, where over 300 flowering time genes, including key regulators, have been identified [18]. These genes are involved in seven major pathways governing flowering: "photoperiod/circadian clock", "vernalization", "aging", "ambient temperature", "hormones", "sugar", and "autonomous" pathways. The key signaling integrator molecule promoting flowering is encoded by the *FLOWERING LOCUS T* (*FT*) gene in leaves. Upon induction, the FT protein migrates from the leaves to the shoot apex, where it activates meristem identity genes [19, 20].Conversely, the product of the *TERMINAL FLOWER1* (*TFL1*) gene functions as an 'anti-florigen', suppressing meristem identity genes [21]. While gene families and pathways controlling flowering time in *A. thaliana* are generally conserved in legumes, three main differences stand out [15, 17, 22, 23]. First, there are variations in the number of gene copies in legumes, with numerous examples of duplication and loss events reflecting the evolutionary history after the divergence of *Arabidopsis* and legume lineages [24]. For instance, legumes possess multiple *FT* genes organized into three subclades and multiple *TFL1* genes [15, 16, 25, 26]. Second, galegoid legume species, such as chickpea, lack *FLC* orthologs, leaving the vernalization response mechanism unknown. However, *FT* genes seem to be major targets of vernalization, similar to *A. thaliana* [17, 25–28]. Third, *CO* orthologous genes in legumes do not seem to play a central role in integrating photoperiod signaling and circadian rhythms, unlike in *A. thaliana* [29, 30].

Traditionally, classical genetic studies have identified four major Mendelian loci that control flowering time in chickpea. Recessive alleles at these loci confer early-flowering [31]. These loci have been designated as *Early flowering1* (*Efl1*) to *Efl4*, with corresponding mutant alleles labeled as *efl1* to *efl4*. The initial identification of these loci occurred in specific lines: ICCV 2 (*Efl1*; [32]), ICC 5010 (*Efl2*; [33]), BGD-132 (*Efl3*; [34]), and ICC 16641 and ICC 16644 (*Efl4*;[31]). Studies have shown that these flowering time genes are non-allelic [31, 34]. In addition, numerous quantitative trait loci (QTLs) associated with flowering time have been identified through linkage analysis, with some predicted to possess minor effects. These QTLs are distributed across various linkage groups (LG), including LG1, LG2, LG3, LG4, LG5, LG6, and LG8, as reported in studies using different parental lines [7, 35–39]. Despite the identification of these major loci and QTLs, the correspondence and characterization of the underlying genes have been limited to date. It has been proposed that the *Elf1* locus corresponds to *CaELF3a*, an ortholog of *Arabidopsis ELF3* mapped on Ca5, although the possibility of other nearby genes contributing to the *Efl1* phenotype cannot be definitively excluded [40]. For the QTL in LG3, the cluster *FTa1*/*a2*/*c* has been identified as the strongest candidate [16].

To deepen our understanding of the genes governing early-flowering phenotypes in chickpea, developing of near-isogenic lines (NILs) emerges as a promising strategy. Pairs of NILs, designed to exhibit variation in specific agronomic traits, have proven invaluable for fine mapping of QTLs and characterizing underlying genes [41]. NILs are distinguished by differences in small genomic sections, effectively minimizing background genetic noise. This plant material facilitates the assessment of allelic variation at both phenotypic and molecular levels, enabling comparisons at genomic or transcriptomic scales. The characteristics of NILs not only provide a focused study of flowering time but also offer accessibility to explore interconnected traits. In chickpea, NILs have been successfully applied in studies on growth habit [42], plant height [43], double/single pod [44, 45]*,* nodulation [46], *Fusarium* wilt resistance [47–49], and flowering time [50].

Advances in next-generation sequencing (NGS) technologies have enabled the generation of large-scale sequencing and genotyping datasets in chickpea, resulting in the creation of valuable genomic resources since the first sequenced genome [1]. One notable achievement is the comprehensive mapping of variation acquired through the sequencing of 3,171 cultivated and 195 wild accessions, alongside phenotypic data, now publicly accessible via the CicerSeq repository [51]. Additional resources like Atlas GEO chickpea complement these datasets, providing a robust foundation for comprehensive investigations into gene function and transcriptional pattern expression in various tissues throughout chickpea development [52]. This extensive dataset serves as a vital resource for genomic and diversity research, facilitating a deeper molecular-level understanding of traits essential for enhancing chickpea cultivation.

In this study, we identified and characterized candidate genes for chickpea flowering through a combined phenotypic and genetic analysis involving re-sequencing of a pair of NILs. Utilizing bioinformatics approaches and public genomic datasets, we identified homologs to flowering-related genes in *A. thaliana* with variants among the NILs, including *ELF3*, and, for the first time in chickpea, *MED16* and *STO/BBX24*. We also analyzed the allelic diversity of these novel genes and their conservation within chickpea diversity. Additionally, transcriptomic data enable us to explore in silico expression profiles for candidate genes in vegetative tissues, such as leaves and the shoot apical meristem, which are crucial for promoting flowering, as well as in early flowering stages.

## Materials and methods

### Plant materials and NIL development

A pair of chickpea NILs distinguished by flowering time was employed: an early-flowering NIL (NF10/82-E) and a late-flowering NIL (NF10/82-L). These NILs were developed from residual heterozygosity in a F_6:7_ recombinant inbred line (RIL) named RIP10–82, derived from the intraspecific cross JG62 x ILC72. This methodology is an alternative to the traditional approach involving consecutive backcrossing followed by self-pollination and is known for its effectiveness in self-pollinated crops like chickpea [53]. The parental line JG62 (syn. ICC4951) is an Indian early-flowering desi landrace (53 days to flowering under long-day conditions, sown in March 2022, in the IFAPA site in Córdoba, Spain; latitude/longitude/altitude: 37º53’N/4º47’W/117 m) maintained by ICRISAT (International Crops Research Institute for the Semi-Arid Tropics). ILC72 (syn. CPAM88) is a late-flowering kabuli type (67 days to flowering under long-day conditions, sown in March 2022, in the IFAPA site in Córdoba, Spain) from the former Soviet Union maintained by ICARDA (International Center for Agricultural Research in the Dry Areas). Descendants from the early-flowering individuals of RIP10–82 consistently showed early-flowering phenotype, while some from late-flowering individuals exhibited segregation for this trait. This observation suggests that early-flowering should be a recessive trait in this context.

**Fig. 1:**
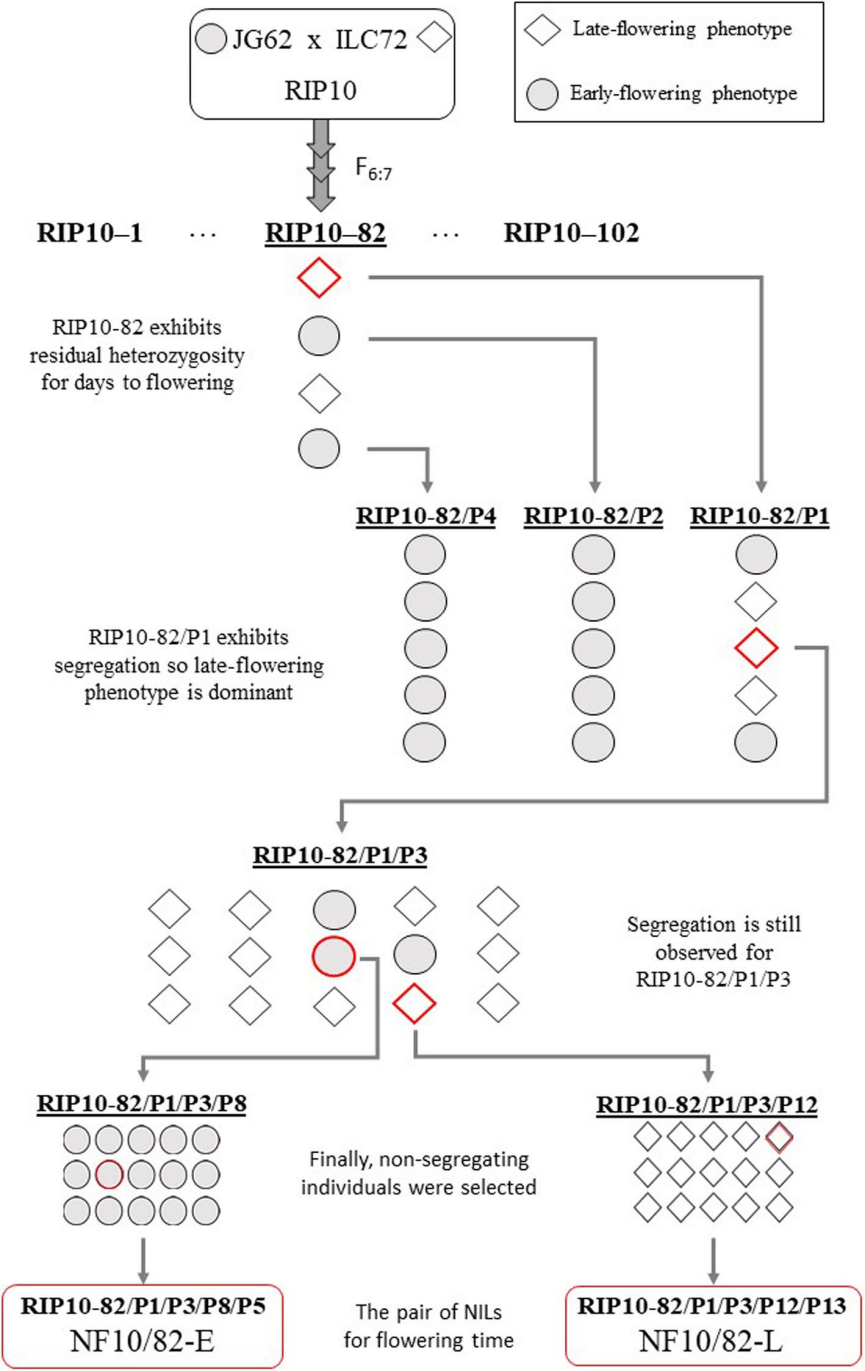
Scheme followed in the present study to develop the pair of NILs for flowering time. NILs were derived from the residual heterozygosity in the recombinant inbred line RIP10-82. The different plants (P) used during development are numbered and represented by symbols based on their phenotype; those selected to produce the NILs are outlined in red

To develop the NILs, seeds from an individual heterozygous plant were collected and sown, designated as RIP10–82/P1 (Fig. 1). Subsequently, a heterozygous descendant for flowering time was selected to proceed with (RIP10–82/P1/P3). Two non-segregating progeny were selfed for both early (RIP10–82/P1/P3/P8) and late-flowering (RIP10–82/P1/P3/P12). One descendant from each one was selfed once more and considered as NILs for this trait: an early-flowering line (RIP10-82/P1/P3/P8/P5, called NF10/82-E) and a late-flowering line (RIP10-82/P1/P3/P12/P13, called NF10/82-L). This means that the NILs were obtained after at least 11 generations of self-fertilization (seven until the RIP10-82 line was obtained and four more thereafter).

### Growth conditions and phenotypic characterization

Phenotypic characterization of the pair of NILs involved the assessment of 15 plants each, sown on March 28, 2022, in the field at the IFAPA site in Córdoba, Spain (latitude/longitude/altitude: 37º53’N/4º47’W/117 m). The plants were arranged in two independent rows, 2.25 m apart. Days to flowering (DTF) were recorded from seedling emergence to the opening of the first flower for each plant. Subsequently, the plants were harvested, dried and phenotyped for six morphological traits: plant weight (PW, g), plant height (PH, cm), internodes per plant (IPP), internode length (IL, cm), total number of branches per plant (BPP) and the number of branches in the first three nodes (BF). Additionally, a branching index (BI) was calculated, defined as the ratio of total branch length to plant length, to normalize differences in general vigor. Statistical significance was assessed using a t-test (*P* < 0.05) in RStudio v.4.2.0.

### DNA extraction and resequencing

Total genomic DNA was isolated from young leaves of individual NF10/82-E and NF10/82-L using the DNeasy Plant Mini Kit (Qiagen) following the manufacturer’s instructions. Resequencing of the two genotypes was conducted using the whole genome shotgun (WGS) approach. DNA samples from both NILs were resequenced by *Centro Nacional de Análisis Genómico* (CNAG-CRG; Barcelona, Spain) using an *Illumina HiSeq2000* instrument with 50x coverage. The data underwent processing with Illumina Sequencing Analysis Viewer, Illumina run specifications, FastQC, and Quality control alignment INS-017. Over 170 million read-pairs were obtained for each genotype.

Variants (SNPs and InDels) were identified against the chickpea reference genome (CDC Frontier genome, assembly ASM33114v1; NCBI). Variants with a read depth < 10 in at least one sample were excluded from consideration. The dataset generated is available in the European Variation Archive (EVA) at EMBL-EBI under accession number PRJEB73790.

### Genetic characterization

The sequence differences detected between the NILs were analyzed, distinguishing between homozygous and heterozygous positions. The identified variants (SNPs and InDels) were filtered, including only those passing all quality criteria or failing to meet only one criterion. These criteria are summarized in the FILTER comments in the VCF v.4.2 file containing the variant sequencing report of the NILs (accession number PRJEB73790; European Variation Archive at EMBL-EBI). Our selection focused exclusively on homozygous variants confidently assigned to chromosomes, determined by the absence of segregation for flowering time observed in the phenotypic data collected at the end of NIL development. Theoretical impact assessment of variants was conducted using snpEff v.4.x [54], categorizing them as modifier, low, moderate, or high impact.

To assess intragenic variants, all variants with an annotation impact other than "intergenic region", "upstream gene variant" or "downstream gene variant" were selected. These variants located in loci were classified by their specific region type as mRNA (coding sequence (CDS), exon or intron), lncRNA, rRNA, snRNA, snoRNA or pseudogene/miscellaneous RNA variant according to the *C. arietinum* GFF data information from NCBI using a custom R script (GitHub/AGR114molecularBreeding/chickpea/SNP_PosType).

The density of variants in chromosomes was visualized using SRplot tools [55]. For protein-coding genes, protein accession was obtained using the *refseqR* package v.1.0.1 [56]. The Gene Ontology Tool Blast2GO v.6.0 [57] was employed to assign GO identities for functional annotation of the protein-coding genes with variants in exons or CDS. The following settings were used: BLASTp against NCBI nr database, *E*-value filter ≤ 10^–3^, HSP length cutoff of 33, maximum 10 BLAST hits per sequence and annotation cutoff of 33. Furthermore, to enhance the annotation ability, InterProScan was conducted, results were merged to GO annotations and plant GO Slim were obtained. An enrichment analysis, calculated via Fisher’s exact test, was performed to compare the functional annotations of the protein-coding genes with variants in exons or CDS against the whole chickpea genome annotation.

### Candidate genes

From the functional annotation, candidate genes were selected based on the enriched GO terms derived from a dataset of 306 flowering-related genes in *A. thaliana*, obtained from the FLOR-ID database [18]. The Go Term Enrichment for Plants tool, available through TAIR and powered by PANTHER [58], was employed for this analysis. Only those child GO Slim terms within each ancestor GO Slim were considered (Additional file 2). Additionally, a reciprocal BLASTp was performed to identify whether any of the candidate genes showed homology to those included in the *A. thaliana* FLOR-ID dataset. A protein–protein interaction and network analysis were performed on specific candidate genes using the STRING (Search Tool for Retrieval of Interacting Genes/Proteins) database (v.12.0, https://string-db.org) to confirm their potential role in flowering time. The “Single Protein by Name” search option was employed against the *C. arietinum* genome with default settings.

### In silico expression analysis

The CDS of the candidate genes were utilized to identify their corresponding matches through reciprocal BLASTn in the chickpea expression atlas during development, available in the NCBI GEO database under the accession GSE147831 [52]. The expression atlas data were then imported, classified and analyzed using a custom R script to convert the FPKM data into TPM, facilitating comparison between different tissues and genes (GitHub/AGR114molecularBreeding/chickpea/GEO). All matched genes were examined for their in silico expression pattern using data from seven different chickpea tissues: young leaf (YL), mature leaf (ML), four stages of flower-bud (FB1–4) and shoot apical meristem (SAM). The heatmap visualization plot for expression level was obtained using SRplot tools with complete-linkage cluster method and Euclidean distance [55].

## Results

### Phenotypic Characterization

**Fig. 2:**
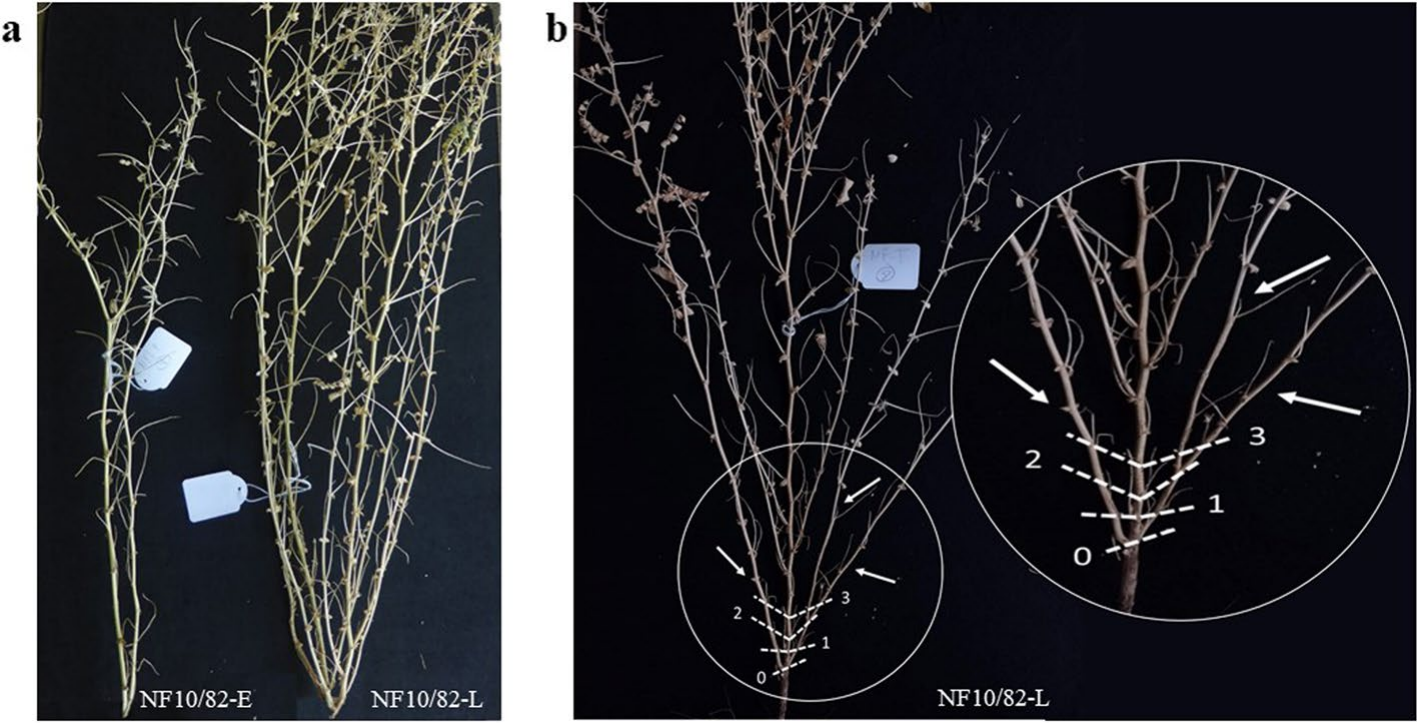
Representative phenotypes of NF10/82-E and NF10/82-L plants grown in the field. **a** Comparison of vegetative biomass characterized by contrasting branching and plant height. **b** NF10/82-L plant characterized by increased branching (more total number of branches and more branches in the initial nodes)

The traits recorded for grown-field NILs are shown in Table 1*.* The difference in flowering time between NILs was approximately 14 days (44.4 ± 2.8 days for NF10/82-E and 58.0 ± 1.1 days for NF10/82-L). NF10/82-E exhibited reduced vegetative biomass, characterized by decreased branching (fewer total branches and fewer branches in the initial nodes) and shorter plant height with fewer internodes (Fig. 2). Nevertheless, its internode length exceeded that of the late-flowering NILs (2.47 ± 0.17 for NF10/82-E vs. 2.23 ± 0.10 for NF10/82-L).

**Table 1:** Phenotypic characterization of the NILs (Mean ± SD)

Genotype	DTF	PW	PH	IPP	IL	BPP	BF	BI
NF10/82-E	44.4 ± 2.8	3.79 ± 1.85	52.5 ± 6.1	22.3 ± 2.6	2.47 ± 0.17	3.91 ± 2.26	0.73 ± 0.65	1.25 ± 0.67
NF10/82-L	58.0 ± 1.1	9.14 ± 2.62	66.6 ± 3.8	30.9 ± 2.4	2.23 ± 0.10	21.5 ± 6.6	2.57 ± 1.02	6.01 ± 1.69
t-test	***	***	***	***	**	***	***	***

Significant difference Student’s t-test (*ns* non-significant, *0.01 < *P* ≤ 0.05, **0.001 < *P* ≤ 0.01, ***P ≤ 0.001). *DTF* Days to flowering, *PW* Plant weight (g), *PH* Plant height (cm), *IPP* Internodes per plant, *IL* Internode length (cm), *BPP* Branches per plant, *BF* Branches in the first three nodes, *BI* Branching index

### Genetic Characterization

The sequencing data confirmed a high degree of similarity between NILs. A total of 393,670,345 positions were read, revealing 120,441 variants (Additional file 1). This indicates that the NILs differ in only 0.03% of positions. For NF10/82-L 209,276 heterozygous positions were detected, and for NF10/82-E, 200,084 for the NF10/82-E, corresponding to observed heterozygosity of 0.053% and 0.051%, respectively. Both lines underwent at least 11 generations of self-fertilization during their development (Fig. 1), so the expected residual heterozygosity is 0.098%, as deduced from Mendelian Genetics for self-fertilizing generations. The observed lower values are reasonable due to prior refreshing processes to maintain seed viability, which may have involved additional self-fertilization generations before obtaining the NILs.

Approximately 64% of the detected variants were successfully mapped to chromosomes (77,170), of which 45,481 met the applied quality criteria (Table 2). Only 15,690 variants were homozygous, with 4,932 being intragenic in 432 loci (HHQ-I variants; Additional file 3). There are 37 variants expected to affect two loci simultaneously.

**Table 2:** Number of variants (SNPs and InDels) and protein-coding genes affected per chromosome in the pair of NILs

Chr	Size (Mb)	Detected Variants	HQ Variants	HHQ Variants	HHQ-I Variants	HHQ-I-C/E Variants	Protein-coding Genes with HHQ-I-C/E
Ca1	48.36	31,790	23,428	12,585	4,185	1,334	165
Ca2	36.63	7,012	3,774	673	134	35	30
Ca3	39.99	5,311	2,095	9	0	0	0
Ca4	49.19	5,371	2,259	23	4	2	2
Ca5	48.17	6,843	2,936	106	17	4	4
Ca6	59.46	12,594	7,507	2,232	575	230	40
Ca7	48.96	6,598	2,625	15	3	1	1
Ca8	16.48	1,651	858	47	14	4	4
	TOTAL	77,170	45,481	15,690	4,932	1,610	246

*HQ* High-quality, *HHQ* Homozygous high-quality, *HHQ-I* Intragenic homozygous high-quality, *HHQ-I-C/E* Intragenic homozygous high quality in CDS or exon of mRNA

Among all HHQ-I variants detected on chromosomes, 1,610 are located in CDS or exons (HHQ-I-C/E variants; 849 in CDS, 758 in exon regions and three in both, depending on DNA strand), affecting 246 protein-coding genes (Table 2). Additionally, there are 176 variants located in non-protein-coding RNA genes, including 17 uncharacterized lncRNA (168 variants), two snRNA (4), one snoRNA (1) and three tRNA (3) (Additional file 4). Finally, 199 variants are positioned in pseudogenes or miscellaneous RNA. Notably, six variants are classified as intragenic, affecting LOC101491595, but in a region devoid of additional features according to the GFF data of *C. arietinum*. This discrepancy is attributed to an error in the annotation of the non-protein-coding transcript XR_003470270.1, as explained by NCBI staff (personal communication). Consequently, XR_003470270.1 has recently been suppressed by NCBI RefSeq staff.

**Fig. 3:**
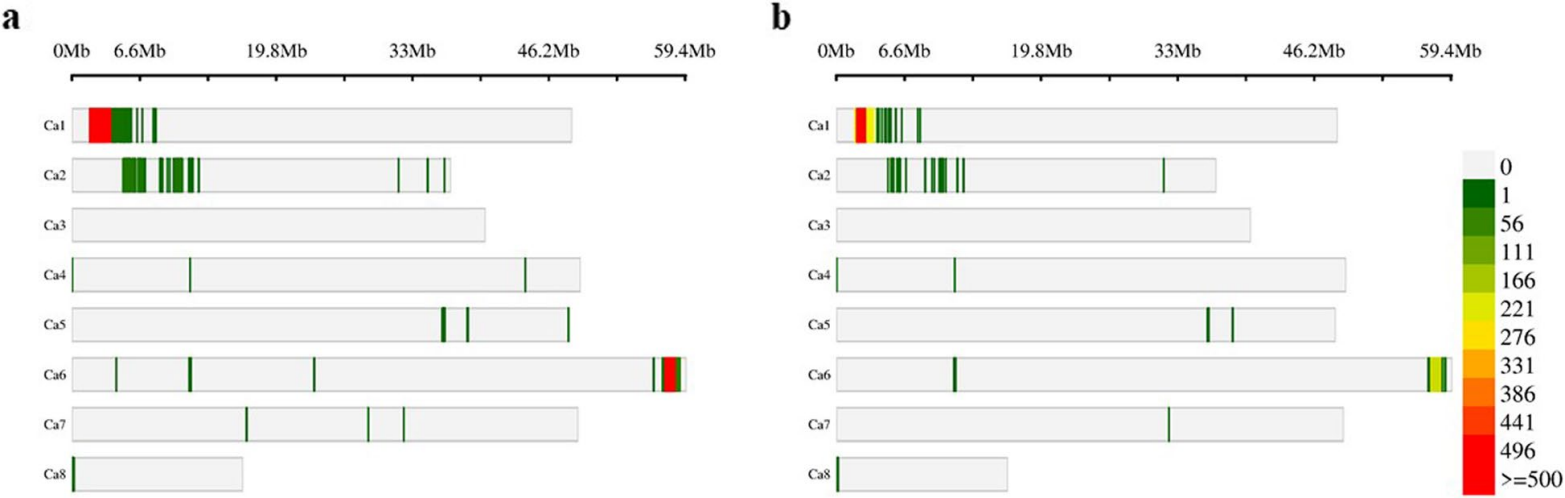
Density in 1 Mb window size over the chickpea chromosomes in the NILs for the **a** 4,932 HHQ-I variants and **b** 1,610 HHQ-I-C/E variants

The distribution of the HHQ-I and HHQ-I-C/E variants did not follow a proportional pattern concerning chromosome size, nor was it uniform along the chromosomes (Fig. 3). Most variants are positioned in a region at the beginning of chromosome 1 (Ca1: 1.78 – 3.15 Mb) and the end of chromosome 6 (Ca6: 57.2 – 58.8 Mb). These are the only two chromosomes with specific regions containing more than 200 variants per 1 Mb window. Notably, chromosome 3 lacks any HHQ-I variant.

**Fig. 4:**
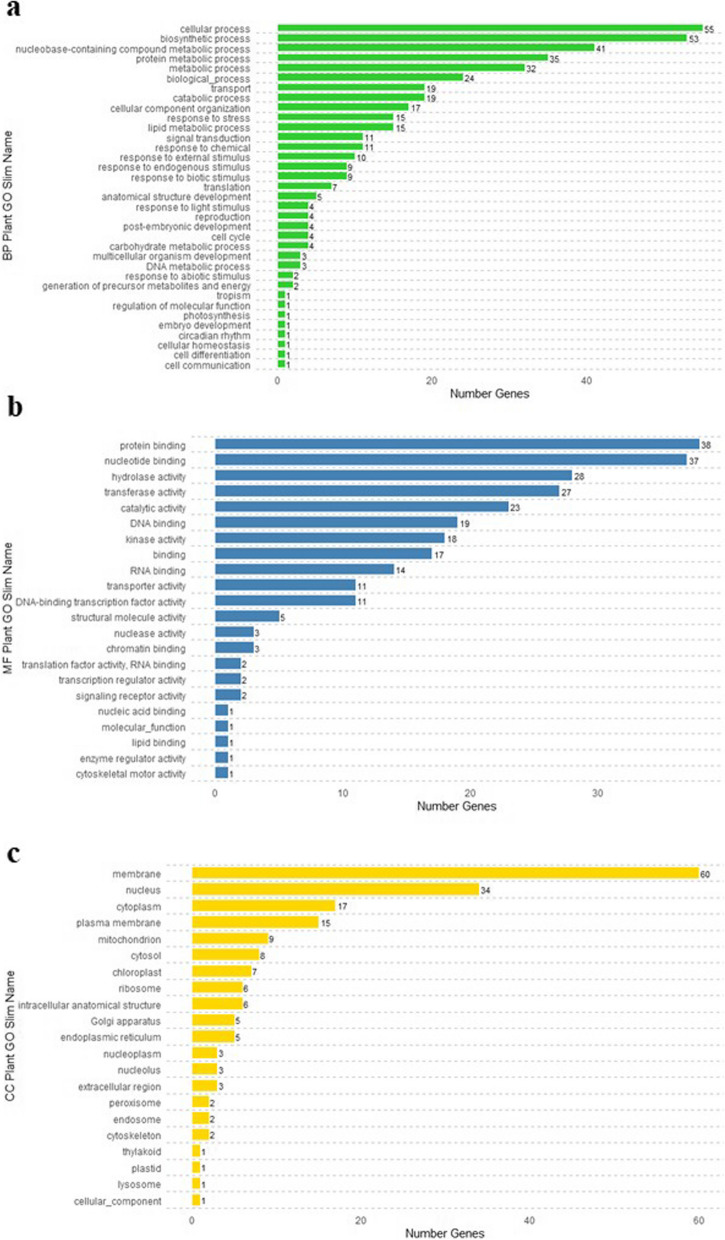
GO Slim terms distribution in the category **a** biological process, **b** molecular function and **c** cellular component for the protein-coding genes affected by HHQ-I-C/E variants in the NILs

Functional annotation using Blast2GO was successfully performed on 216 out of the 246 coding genes affected by HHQ-I-C/E variants (Additional file 5). The distribution of GO Slim terms among these protein-coding genes across the ontologies of “molecular function”, “biological process”, and “cellular component” (Fig. 4) revealed no enrichment compared to the entire chickpea annotated genome using Fisher's exact test (*P* < 0.05).

### Candidate Genes

Based on GO Slim enrichment analysis in *A. thaliana* for the FLOR-ID set of flowering-related genes, 146 genes affected by HHQ-I-C/E variants have these enriched GO Slim terms (Additional file 6). Among them, four genes are found to be homologous to those in the FLOR-ID *A. thaliana* dataset according to the reciprocal BLASTp results. These genes are LOC101515142, LOC101489432 (also known as *CaELF3a*), LOC101499101 and LOC101507442 (Table 3).

**Table 3:** Genes affected by HHQ-I-C/E variants that are homologous to four genes included in the *A. thaliana* FLOR-ID dataset

*C. arietinum* ID NCBI	Homologous *A. thaliana* ID TAIR	Number HHQ-I-C/E Variants	Variant Position	Ref (0)	Alt (1)	Variant Impacts	NF10/82-L	NF10/82-E
**LOC101515142** mediator of RNA polymerase II transcription subunit 16-like (Ca1: 2,285,592 – 2,298,911, complement)	AT4G04920	14 (+ 80 in intron regions)	2,285,696	C	CAT	3_prime_UTR_variant [MODIFIER], non_coding_transcript_variant [MODIFIER]	0/0	1/1
			2,285,879	G	A	3_prime_UTR_variant [MODIFIER], non_coding_transcript_variant [MODIFIER]	0/0	1/1
			2,286,112	G	GA	3_prime_UTR_variant [MODIFIER], non_coding_transcript_variant [MODIFIER]	0/0	1/1
			2,286,256	A	T	3_prime_UTR_variant [MODIFIER], non_coding_transcript_variant [MODIFIER]	0/0	1/1
			2,286,460	C	T	3_prime_UTR_variant [MODIFIER], non_coding_transcript_variant [MODIFIER]	0/0	1/1
			2,286,488	A	G	3_prime_UTR_variant [MODIFIER], non_coding_transcript_variant [MODIFIER]	0/0	1/1
			2,288,143	C	T	synonymous_variant [LOW], non_coding_transcript_variant [MODIFIER]	0/0	1/1
			2,291,280	T	G	synonymous_variant [LOW], non_coding_transcript_variant [MODIFIER]	0/0	1/1
			2,292,486	A	C	synonymous_variant [LOW], non_coding_transcript_variant [MODIFIER]	0/0	1/1
			2,292,528	G	A	synonymous_variant [LOW], non_coding_transcript_variant [MODIFIER]	0/0	1/1
			2,294,335	A	G	synonymous_variant [LOW], non_coding_transcript_variant [MODIFIER]	0/0	1/1
			2,298,490	G	T	synonymous_variant [LOW], 5_prime_UTR_variant [MODIFIER], non_coding_transcript_variant [MODIFIER]	0/0	1/1
			2,298,571	CTCTTCT	C	disruptive_inframe_deletion [MODERATE], 5_prime_UTR_variant [MODIFIER], non_coding_transcript_variant [MODIFIER]	0/0	1/1
			2,298,634	T	A	synonymous_variant [LOW], 5_prime_UTR_variant [MODIFIER], non_coding_transcript_variant [MODIFIER]	0/0	1/1
**LOC101489432** protein EARLY FLOWERING 3a (Ca5: 36,011,384 – 36,016,600, complement)	AT2G25930	1	36,016,064	ATCATCATCTTC	A	frameshift_variant [HIGH], non_coding_transcript_exon_variant [MODIFIER], non_coding_transcript_variant [MODIFIER]	0/0	1/1
**LOC101499101** B-box zinc finger protein 24 (Ca6: 57,549,424 – 57,552,323, complement)	AT1G06040	1	57,549,449	T	A	3_prime_UTR_variant [MODIFIER], non_coding_transcript_variant [MODIFIER]	1/1	0/0
**LOC101507442** B3 Domain- containing transcription factor VRN1-like (Ca6: 57,717,926 – 57,721,229)	AT3G18990	1	57,720,344	C	T	synonymous_variant [LOW], non_coding_transcript_variant [MODIFIER]	0/0	1/1

LOC101515142 (Ca1: 2,285,592 – 2,298,911, complement) is annotated as “mediator of RNA polymerase II transcription subunit 16-like” (*MED16*), an homologue of the *A. thaliana MED16/SFR6* gene, which encodes a component of the Mediator complex involved in various aspects of gene expression regulation [59]. In chickpea, a second homologue is present on Ca6 (LOC101501202, Ca6: 16,660,218 – 16,679,432) with no detected variants between NILs. LOC101515142 is affected by 94 variants, of which 14 affect exon or CDS regions (Table 3). Most alternative variant alleles are found in NF10/82-E, with only one detected in NF10/82-L (a 21 bp deletion located in an intron at Ca1: 2,297,103). This locus encodes six different isoforms, affected by three variants in CDS with varying impacts. For instance, NF10/82-E has a 6 bp deletion (Ca6: 2,298,571) impacting two isoforms moderately (loss of two Glu), while two transcriptional isoforms are affected in the 5′ UTR (Additional file 7. Fig. S1a). However, the other 11 HHQ-I-C/E variants affect all isoforms equally with low or modifier theoretical impacts. The high number of detected variants affecting this locus could have implications for its functional activity.

LOC101489432 (*CaELF3a*, Ca5: 36,011,384 – 36,016,600, complement) is one of the two homologs of *A. thaliana ELF3* identified in legumes, previously reported to regulate the circadian clock and influence flowering in chickpea [40]. In this study, an 11 bp deletion at Ca5: 36,016,064 was detected in NF10/82-E. This deletion is predicted to affect the first exon of *CaELF3a*, resulting in six missense amino acids followed by a premature stop codon. This alteration reduces the protein length from 699 to 13 amino acids (Additional file 7. Fig. S1b), indicating a significant impairment in its functionality.

Finally, two loci at the end of Ca6 are also affected by HHQ-I-C/E variants. LOC101499101 is a B-box finger protein homolog of *STO/BBX24*, known to link the *FRI/FLC* and photoperiod/circadian clock pathways, influencing flowering time in *A. thaliana* [60]. LOC101507442 is a *VRN1*-*like* transcription factor containing a B3 domain, encoding a DNA-binding protein involved in the vernalization pathway that represses *FLC* expression, promoting flowering [61]. Both loci are affected by only one SNP with modifier or low theoretical impact. LOC101499101 has a SNP affecting the 3′ UTR (Additional file 7. Fig. S1c), while LOC101507442 has a SNP located in the third exon, influencing its two potential protein isoforms as a synonymous variant (Additional file 7. Fig. S1d).

**Fig. 5:**
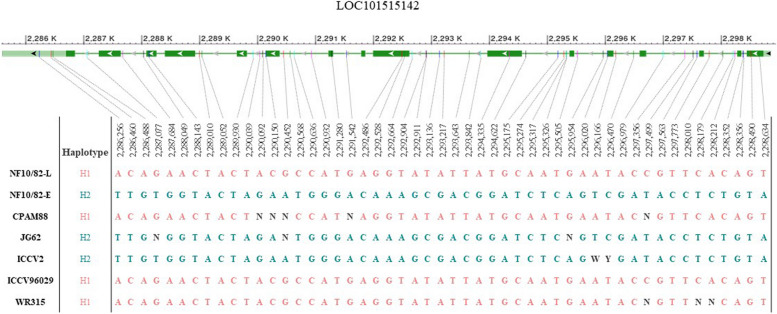
Conservation of the LOC101515142 haplotypes detected in the NIL pair across some cultivated *C. arietinum* accessions (data obtained from 51 SNPs registered in the CicerSeq pangenome public repository). The NF10/82-L haplotype (H1) is conserved in 2,219 accessions (~ 70%), while the NF10/82-E (H2) is present in 749 (23.6%)

The 11 bp deletion in *CaELF3a* is distributed across a small proportion of chickpea germplasm, with only two haplotypes identified that specifically differ in this deletion [40]. To gain insights into the genetic variability and conservation of the variants detected in LOC101515142, LOC101499101 and LOC101507442 along chickpea diversity, we analyzed different accessions represented in the public repository CicerSeq, which contains SNP information for cultivated chickpea [51]. Among the 94 variants detected in LOC101515142 in the pair of NILs, 68 are SNPs, with 51 positions registered in CicerSeq. The contrasting haplotypes for LOC101515142 detected in NILs are highly conserved across the 3,171 cultivated accessions for *C. arietinum* registered in the pangenome (Fig. 5 and Additional file 8). The NF10/82-L haplotype is conserved in approximately 70% of the accessions (H1), while the NF10/82-E is present in about 23.6% (H2). Interestingly, ~ 3.8% of accessions have the NF10/82-L haplotype except for one SNP variant located in Ca1: 2,295,317 (in the intron region of LOC101515142*;* H3), and 2% of accessions have an intermediate haplotype (36/51 SNPs like NF10/82-L haplotype; H4). For the SNPs in LOC101499101 (T/A) and LOC101507442 (C/T), the reference alleles are the majority (82.2% T/ 9.4% A and 83.7% C/ 7.3% T, respectively) (Additional file 9).

**Fig. 6:**
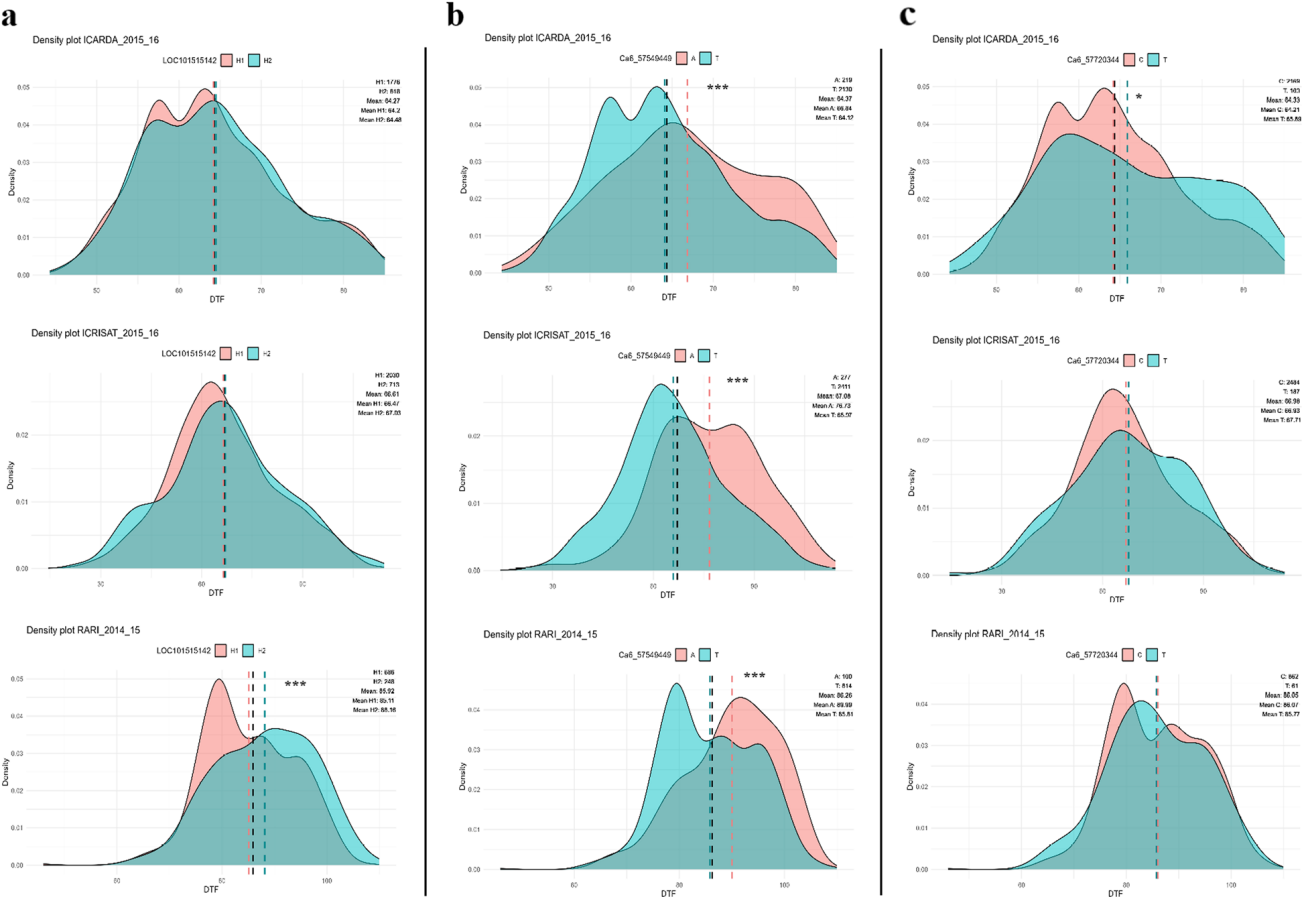
Density plot of cultivated chickpea accessions distribution based on days to flowering (DTF) in three locations (ICARDA 2015/16; ICRISAT 2015/16; RARI 2014/15) according to **a** LOC101515142 haplotype, **b** LOC101499101 SNP (Ca6: 57,549,449), and **c** LOC101507442 SNP (Ca6: 57,720,344). The DTF data were acquired from the public repository CicerSeq. Vertical lines represent the global mean (black) and the means for each group (salmon and turquoise). Significant differences were assessed using Student’s t-test (ns: non-significant, *0.01 < *P* ≤ 0.05, **0.001 < *P* ≤ 0.01, ***P ≤ 0.001). The number of individuals taken into account for each location/season depending on the SNPs they present is indicated in the upper left corner of each of the plots. Additional data for other locations can be found in Additional file 10

The DTF data for chickpea accessions, available in the public repository CicerSeq across six locations and two seasons (excluding the IIPR location, which provided data for only one season), were analyzed according to the SNPs present in LOC101515142 haplotype, LOC101499101 and LOC101507442 (Additional file 9). Accession density distribution plots were obtained for each group (Fig. 6 and Additional file 10). Significant differences in DTF were observed in accessions with contrasting haplotypes of LOC101515142 across three different locations/seasons. Conversely, in other locations, the DTF distribution among different lines within the two groups of accessions with contrasting haplotypes was similar. This pattern is also observed for the SNP in LOC101507442, where significant differences were detected in ICARDA 2015/16. In contrast, the SNP located in LOC101499101 exhibited significant differences in DTF across all locations/seasons, except for RARI 2015/16. These consistent differences suggest that LOC101499101 could influence flowering time across chickpea diversity, while also indicating a potential contribution of LOC101515142.

To elucidate the role of LOC101515142 and LOC101499101 in flowering, we utilized the STRING database to explore their protein–protein interactions (PPI). The PPI network for LOC101515142 (“mediator of RNA polymerase II transcription subunit 16-like”) is primarily associated with components of the Mediator complex (PPI enrichment *P* < 1.0 × 10^–16^. Additional File 11. Fig. S1a). Functional enrichment network analysis identified the biological process GO term GO:2000028 “regulation of photoperiodism, flowering” as one of the most significant term, associated with LOC101515142 and LOC101491075 (“mediator of RNA polymerase II transcription subunit 18-like”). The PPI network for LOC101499101 (“B-box zinc finger protein 24”) revealed interactions with LOC101510767 (“E3 ubiquitin-protein ligase COP1”) and LOC101512390 (“transcription factor HY5-like”), with a PPI enrichment *P* = 0.212 (Additional File 11. Fig. S1b). All the enriched GO terms for biological processes are related to light response and photomorphogenesis, suggesting a potential association with flowering.

### In silico expression analysis

A total of 132 CDS from the selected 146 protein-coding genes, based on their functional annotation, were unambiguously matched with sequences in the GEO dataset. The TPM data for each transcript ID and their corresponding gene ID can be found in Additional file 12.

**Fig. 7:**
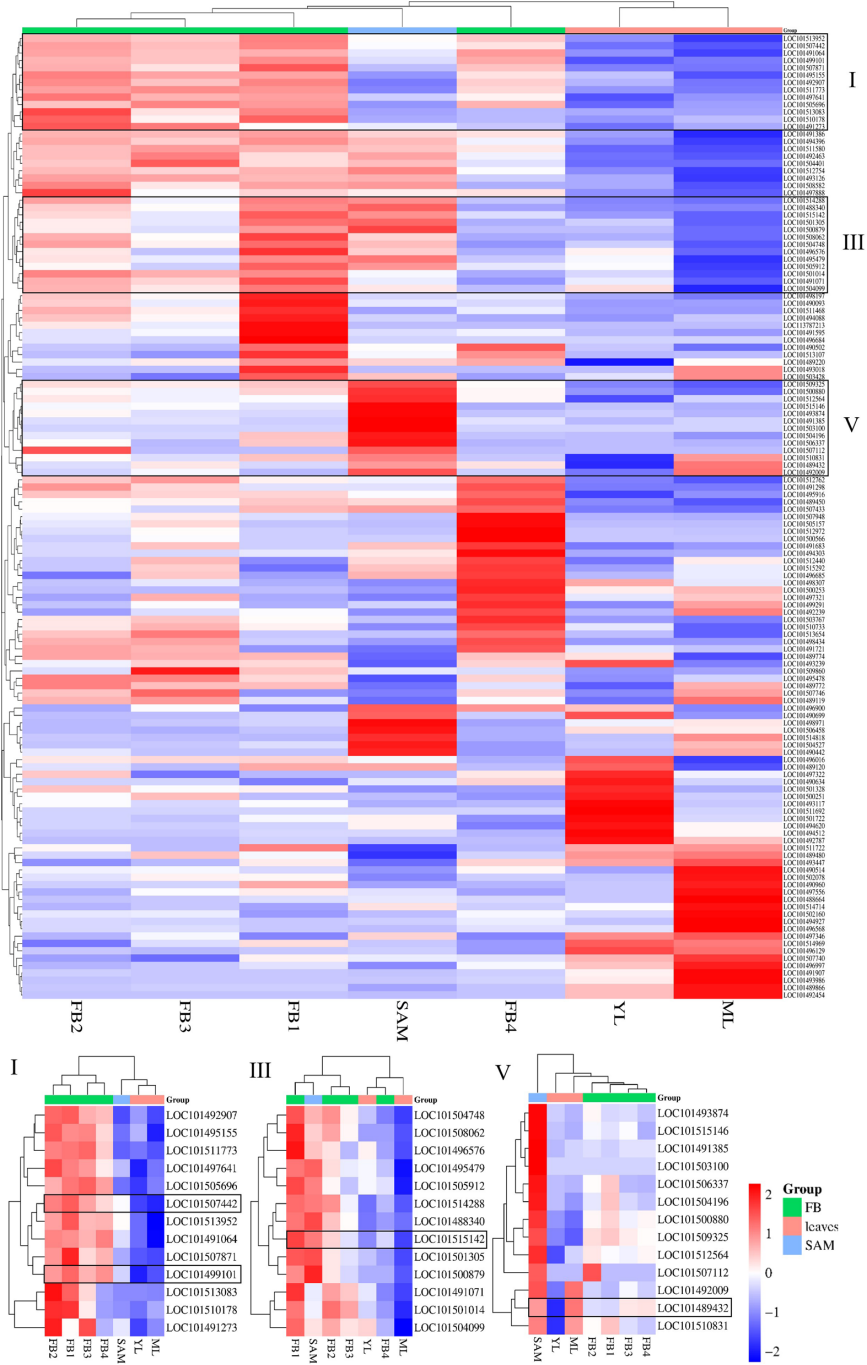
The heatmap expression level of the 132 matched genes with HHQ-I-C/E variants in NILs. The four genes homologous to those included in the FLOR-ID *A. thaliana* dataset appear in three clusters. LOC101499101 and LOC101507442 appear in Cluster I. LOC101515142 appears in Cluster III, close to those genes, whereas LOC101489432 shows the most different expression profiles (Cluster V). Tissue Samples: YL, young leaf; ML, mature leaf; SAM, shoot apical meristem; FB (1 – 4), flower bud (different development stages 1 to 4). The TPM data for each transcript ID and their corresponding gene ID can be found in Additional file 12

The TPM level heatmap categorized LOC101515142, LOC101489432, LOC101499101 and LOC101507442 into three distinct clusters (Fig. 7). LOC101499101 and LOC101507442 (both situated at the end of Ca6) show a similar expression pattern, closely grouped in the same subcluster, characterized by genes with higher expressions at all FB stages (Cluster I). LOC101515142 (Ca1: 2,285,592 – 2,298,911, complement) is in a neighboring cluster (Cluster III) with lower expression levels at the end of the FB stage (FB3 and FB4), but higher levels in SAM. Finally, LOC101489432 (Ca5: 36,011,384 – 36,016,600, complement) shows the most different expression profile, falling into a cluster with high expression levels in SAM and low expression in FB tissues (Cluster V). This locus is somewhat isolated from other genes in its cluster due to its lower expression level in YL and higher level in ML. The detailed description of co-expressed genes for these four genes can be found in Table 4.

**Table 4:** Co-expressed genes associated with the four genes affected by HHQ-I-C/E variants (highlighted in bold) homologous to genes included in FLOR-ID

Cluster	Gene ID	Variants	GO Slim Name	Gene Description
I	LOC101492907	8	P: lipid metabolic process	enoyl-CoA delta isomerase 2, peroxisomal-like
I	LOC101495155	33	F: hydrolase activity	GDSL esterase/lipase At5g45920
I	LOC101511773	1	F: hydrolase activity	ATP-dependent zinc metalloprotease FTSH 4, mitochondrial-like
I	LOC101497641	8	F: protein binding	heterogeneous nuclear ribonucleoprotein U-like protein 1
I	LOC101505696	8	P: response to biotic stimulus; P: response to external stimulus; P: response to stress	putative disease resistance protein At3g14460
**I**	**LOC101507442**	**1**	**F: DNA binding**	**B3 domain-containing transcription factor VRN1-like**
I	LOC101513952	14	P: response to chemical; P: response to endogenous stimulus; P: biosynthetic process; P: signal transduction; F: DNA binding	auxin response factor 2
I	LOC101491064	72	P: biosynthetic process; F: DNA-binding transcription factor activity; F: DNA binding	dof zinc finger protein DOF 4.7-like
I	LOC101507871	1	F: protein binding	pentatricopeptide repeat-containing protein At4g20740-like
**I**	**LOC101499101**	**1**	**P: post-embryonic development; P: response to light stimulus; P: biosynthetic process**	**B-box zinc finger protein 24**
I	LOC101513083	10	P: biosynthetic process; F: DNA-binding transcription factor activity	uncharacterized LOC101513083
I	LOC101510178	6	P: biosynthetic process; F: DNA binding	homeobox-DDT domain protein RLT1
I	LOC101491273	4	P: response to chemical; P: response to endogenous stimulus; P: biosynthetic process; P: signal transduction; F: DNA-binding transcription factor activity; F: DNA binding	ethylene-responsive transcription factor 1B
III	LOC101504748	1	P: biosynthetic process	spermidine synthase 1
III	LOC101508062	19	F: kinase activity; F: protein binding	protein STRUBBELIG-RECEPTOR FAMILY 3-like
III	LOC101496576	29	P: biosynthetic process; F: DNA binding	TATA box-binding protein-associated factor RNA polymerase I subunit B
III	LOC101495479	37	P: biosynthetic process	THO complex subunit 7A-like
III	LOC101505912	1	F: kinase activity	protein kinase PINOID-like
III	LOC101514288	37	P: biosynthetic process	splicing factor U2af large subunit B-like
III	LOC101488340	102	P: biosynthetic process; F: protein binding	proteinaceous RNase P 2
**III**	**LOC101515142**	**94**	**P: biosynthetic process**	**mediator of RNA polymerase II transcription subunit 16-like**
III	LOC101501305	1	P: signal transduction	14–3-3-like protein C
III	LOC101500879	41	P: biosynthetic process; F: protein binding	pre-mRNA-splicing factor SYF1
III	LOC101491071	41	F: protein binding	phospholipase A-2-activating protein
III	LOC101501014	1	F: kinase activity; F: protein binding	probable inactive leucine-rich repeat receptor-like protein kinase At3g03770
III	LOC101504099	1	P: biosynthetic process; F: hydrolase activity	ribosome biogenesis protein BMS1 homolog
V	LOC101493874	15	P: response to chemical; P: response to stress; F: protein binding	E3 ubiquitin-protein ligase RMA1H1-like
V	LOC101515146	1	F: chromatin binding	uncharacterized LOC101515146
V	LOC101491385	4	F: hydrolase activity	non-cyanogenic beta-glucosidase-like
V	LOC101503100	43	P: response to chemical; P: response to endogenous stimulus; P: signal transduction; F: transporter activity	lysine histidine transporter-like 8
V	LOC101506337	1	P: biosynthetic process; F: hydrolase activity	U4/U6.U5 tri-snRNP-associated protein 2-like
V	LOC101504196	2	P: biosynthetic process; F: DNA-binding transcription factor activity; F: DNA binding	ethylene-responsive transcription factor 12
V	LOC101500880	1	P: biosynthetic process; F: DNA-binding transcription factor activity; F: DNA binding	dof zinc finger protein DOF5.3-like
V	LOC101509325	2	P: response to chemical; P: response to endogenous stimulus; P: signal transduction	two-component response regulator ARR17
V	LOC101512564	86	F: hydrolase activity	allantoinase
V	LOC101507112	1	P: biosynthetic process	SAC3 family protein B
V	LOC101492009	1	P: response to chemical; P: response to endogenous stimulus; P: signal transduction; P: response to stress	protein TIFY 5A
**V**	**LOC101489432**	**1**	**P: post-embryonic development; P: response to light stimulus; P: reproduction**	**protein EARLY FLOWERING 3a**
V	LOC101510831	3	P: response to stress; P: DNA metabolic process	helicase-like transcription factor CHR28

In Cluster I, LOC101513952 (*CaARF2*), an auxin response factor protein (ARF), is notable. The ARF family members are core to auxin signaling, with important functions as regulators of plant growth and developmental processes [62, 63]. NF10/82-E exhibits 14 variants affecting this locus, six of which are located in exon or CDS regions with moderate and low theoretical impacts. LOC101491064,, encoding a DNA-binding one zinc finger (DOF) protein, also stands out in this cluster. DOF transcription factors are involved in various fundamental processes in plants, including responses to light and phytohormones, as well as roles in seed maturation or germination [64]. For this locus, a total of 72 variants were detected in NF10/82-E with 13 located in exon or CDS regions. Additionally, LOC101491273, an ethylene-responsive transcription factor (ERF), is affected by four HHQ-I-C/E variants, one of which is predicted to have a moderate impact as a missense variant. In Cluster III, no other gene apart from LOC101515142 seems to be prominent for flowering.

Finally, LOC101492009 and LOC101510831 show the most similar expression pattern with *CaELF3a* in Cluster V. LOC101492009 encodes the TIFY5A protein, and LOC101510831 encodes a helicase-like transcription factor CHR28, both with the stress response GO Slim term. Moreover, Cluster V includes LOC101504196 (ethylene-responsive transcription factor 12) with two SNPs and LOC101500880 (dof zinc finger protein DOF5.3-like) with a SNP in 3′ **UTR**. Thus, two members of the ERF family and two members of the DOF family cluster with the genes homologous to those found in the FLOR-ID *A. thaliana* dataset.

## Discussion

Near-isogenic lines (NILs) provide a unique advantage by confining genetic variation to specific regions of the genome while preserving genetic identity elsewhere. In this study, we characterized a pair of NILs exhibiting contrasting flowering times, aiming to discern not only major but also minor genes contributing to this complex process.

Phenotyping of both NILs revealed significant differences across various morphological traits, including a notable contrast in DTF (Table 1). This implies that genetic distinctions between the two NILs extend beyond the control of flowering initiation and influence a range of diverse characteristics. The association between flowering and multiple shoot architecture traits has been documented in various legume species, including chickpea [16, 35, 65–71]. Several instances of legume mutants, characterized by alterations in specific flowering-related genes, exhibit variations in morphological features such as changes in branching patterns and internode length [26, 30, 70]. In the case of the studied NILs, phenotype differences could arise from the action of several independent genes or the pleiotropic effects of a single or a few genes. Nevertheless, the substantial differences in DTF observed (14 days) suggest additive effects from more than one locus.

The sequencing data from the pair of NILs revealed a 99.97% identity of the read positions, with variations mainly observed in specific regions, as expected [41]. This level of genomic identity is consistent with values reported in other legume studies involving NILs, including chickpea, where identities range between 90 – 99% [50, 72, 73]. The observed residual heterozygosity for each NIL is ~ 0.05%, falling below the theoretical 0.098% expected for 11 generations of self-fertilizing lines. Nevertheless, this aligns with values reported for other NILs [74] and closely resembles the residual heterozygosity found in cultivated chickpea. According to data reported by Varshney et al. [51], the detected residual heterozygosity for SNPs ranged from 0.024% (0.013% – 0.050%) for cultivar lines to 0.033% (0.011% – 0.078%) for landrace lines and 0.033% (0.009% – 0.073%) for breeding lines, relative to the total sequenced positions (533.36 Mb; Additional file 13). It is important to note that these estimations do not encompass other variations, such as InDels, suggesting that the actual heterozygosity may be higher. Therefore, the pair of NILs developed in our study appears to be suitable plant material, embodying the characteristics of near-isogenic lines, and providing a valuable resource for further genetic and functional studies in chickpea research.

The comparison between positions sequenced in the pair of NILs revealed 15,690 homozygous variants (SNPs and InDels) mapped to chromosomes that pass all quality criteria or fail to meet only one criterion (Table 2). Of these, 4,932 variants are intragenic (HHQ-I), with the highest density observed at the beginning of chromosome 1 and the end of chromosome 6 (Fig. 3 and Additional file 3). Notably, no HHQ-I variants were detected on chromosome 3, where QTLs have been reported several times, and genetic variants in the *FTa1/a2/c* cluster seem to play an important role in relaxing the environmental constraints on flowering, permitting early-flowering in long-day legumes [15, 16]. Thus, differences in flowering time in the pair of NILs do not appear to be related to chromosome 3.

A total of 1,610 variants were identified within exons or CDS (HHQ-I-C/E), affecting 246 protein-coding genes. However, functional annotation against the chickpea genome annotation did not reveal any enrichment of GO Slim terms (Fig. 4). To deepen our analysis, we selected 146 of these as candidate genes, guided by enriched GO Slim terms related to flowering obtained from the model plant *A. thaliana* (Additional file 6). Significantly, four candidate genes showed homology to *A. thaliana* FLOR-ID genes dataset (Table 3). One of them, LOC101507442 (Ca6: 57,717,926 – 57,721,229), a B3 domain-containing transcription factor *VRN1-like*, is affected only by a SNP located in CDS with low impact as a synonymous variant. The analysis of the public repository CicerSeq phenotype data indicates that this SNP is not associated with DTF in chickpea germplasm (Fig. 6 and Additional file 10).

LOC101515142 (Ca1: 2,285,592 – 2,298,911, complement) is a homologue of the *A. thaliana MED16/SFR6* gene, encoding a component of the Mediator complex. This complex plays a pivotal role in regulating RNA polymerase II-dependent gene expression. It serves as a large and dynamically variable multisubunit protein complex that recruits transcription factors to specific gene sites, promoting or repressing transcription initiation and elongation through protein–protein interaction modules [59, 75–77]. The Mediator is highly conserved across eukaryotes, with only four out of the 34 Mediator subunits described in *Arabidopsis* being plant-specific subunits; 25 subunits, including *MED16,* are structurally conserved [59]. *MED16* is part of the tail module of the Mediator complex with functions in both abiotic and biotic stress pathways. Initially identified as *SENSITIVE TO FREEZING 6* (*SFR6*) due to its role in cold acclimation [78–80], *MED16* is also involved in the regulation of iron homeostasis [81] and salicylic acid- and jasmonate-mediated defense response [82, 83]. Loss of *MED16* function disrupts transcriptional outputs beyond low-temperature gene regulation, affecting the expression of genes involved in the photoperiod flowering time pathway and circadian clock. This disruption can lead to a late-flowering phenotype in long days [84].

To our knowledge, no flowering-time-related function for *MED16* has been described in legumes. A recent study in *Medicago truncatula* Gaertn. detected a mutation in a *MED16* homologue (LOC25493186, MtrunA17_Chr4g0047551), denoted as *MED16A* by the authors, which suppresses nodulation and increases arbuscular density [85]. However, a comparison through BLASTp against *C. arietinum* RefSeq_Protein database reveals that *MED16A* seems to be the homologue to LOC101501202 (Ca6: 16,660,218—16,679,432), with no variants between NILs. Thus, LOC101515142, affected by 94 variants in this study, seems to be the homologue of *MED16B* (LOC11424919, MtrunA17_Chr2g0281921) (Additional file 14).

While the specific involvement of *MED16* in flowering time remains unexplored in legumes, recent investigations have shed light on the importance of the Mediator complex in this process. Notably, a recent study in pea highlighted the role of other Mediator complex subunits, specifically orthologs of CYCLIN-DEPENDENT KINASE 8 (CDK8) and CYCLIN C1 (CYCC1), components of the CDK8 kinase module, in promoting flowering and maintaining normal reproductive development [70]. Moreover, in chickpea, a recent study identified the role of two Mediator subunit genes, namely *CaMED23* and *CaMED5b*, and their naturally derived haplotypes, in regulating plant height [43]. These findings underscore the potential importance of the variability within Mediator complex in influencing various traits critical for yield improvement.

Based on the nomenclature used in these previous legume studies focusing on the Mediator complex [43, 70, 85], we propose that LOC101515142 is *CaMED16b*. The identified SNPs in *CaMED16b* appear to form contrasting haplotypes, showing high conservation across cultivated chickpea germplasm (Fig. 5 and Additional file 8). However, significant differences in DTF among accessions with these contrasting haplotypes were observed in only three out of eleven different locations/seasons (Fig. 6 and Additional file 10). Although the PPI network analysis of *CaMED16b* reveals that “regulation of photoperiodism, flowering” is one of the most significantly enriched GO term (Additional file 11), further investigation is required to fully comprehend its functional role in flowering and its contribution to DTF in chickpea.

*CaELF3a* (Ca5: 36,011,384 – 36,016,600, complement) is one of the two homologs of *A. thaliana ELF3* identified in legumes [40, 86]. This gene is a major component of the Evening complex (EC) with *ELF4* and *LUX* within the circadian clock. The EC is not only directly involved in clock function, but also plays a key role in various developmental processes by interacting with other genes, such as *PIF4* or *GI*, thus regulating photoperiodic flowering and hypocotyl elongation in *A. thaliana* [87–90].

In this study, an 11 bp deletion in the first exon of *CaELF3a* was identified in the NF10/82-E line. This deletion is predicted to cause a frameshift, reducing the encoded protein from 699 to 13 amino acids (Additional file 7. Fig. S1b). This deletion was previously reported by Ridge et al. [40] in the ICCV96029 line. It is noteworthy that the 11 bp constituting the deletion are followed by 10 bp that are identical to them. This sequence similarity may have facilitated the natural occurrence of the deletion at this specific position within the gene (Additional file 7. Fig. S1b). In fact, Ridge et al. resequenced the entire *CaELF3a* gDNA in 109 lines and only found this sequence polymorphism [40]. The presence of the deletion in homozygosity is associated with early-flowering in chickpea, representing the recessive allele. This aligns with the observation that the late-flowering phenotype was dominant in the developmental process of the pair of NILs used in this study **(**Fig. 1**)**. Interestingly, *ELF3* acts as a negative regulator of flowering [90], so loss-of-function mutations in this locus are predicted to result in early-flowering phenotype as we observed in the pair of NILs.

Mutations in *ELF3* orthologs are also associated with early-flowering and reduced branching in other galegoid legumes, such as pea and lentil [69], a morphological trait also observed for the NF10/82-E line in this study. However, although *CaELF3a* appears to have a significant effect on flowering time and other related traits, not all of the phenotypic differences detected between the pair of NILs should be assigned to it. Other genes may likely contribute comparable positive effects on flowering time, as expected with ICCV96029 [40].

In LOC101499101 (Ca6: 57,549,424 – 57,552,323, complement), we identified a SNP located in the 3′ UTR. This locus shares homology with the B-box finger protein of *A. thaliana* STO/BBX24, known for its role in connecting the *FRI/FLC* and the photoperiod/circadian clock pathway, ultimately influencing flowering time in this species [60]. The 3′ UTR of mRNA is recognized for its involvement in transcriptional control and protein targeting, affecting various physiological processes in plants, such as flowering and stress tolerance [91, 92]. Specifically, different mechanisms of 3′ RNA processing have been investigated for their relevance to flowering time, with a focus on the *FLC* gene in *A. thaliana* [93, 94]. Furthermore, a study highlighted the role of post-transcriptional regulation in controlling flowering time through repressed *SOC1* activity in a 3′ UTR-dependent manner in *A. thaliana* [95]. Polymorphisms in the UTR and intronic regions have also been associated with higher expression of an *FT5a* allele causing early-flowering in soybean [96].

While the effects of a single SNP in UTRs may not be as pronounced as those in CDS, its association with flowering time should not be dismissed. For example, a SNP in the 3′ UTR of *M. truncatula FTa1* was significantly correlated with latitudinal variation, reflecting differences in photoperiod and temperature in its distribution across the Mediterranean region [97]. Notably, the analysis of accession density distribution plots based on the allele of the SNP reveals significant differences in DTF across all locations/seasons registered in the public repository CicerSeq, except for one (Fig. 6 and Additional file 10). Moreover, the PPI network analysis indicates enriched GO terms related to light response, further suggesting a role in flowering (Additional file 11).Therefore, the T → A transversion detected in LOC101499101 could influence DTF differences in the NILs, implying a plausible association of the SNP with flowering time in the chickpea germplasm.

The in silico expression analysis was conducted to further characterize the 146 candidate protein-coding genes affected by HHQ-I-C/E in the pair of NILs. The purpose of this analysis was to gain insights into the transcription profiles in vegetative tissues crucial for flowering promotion (leaves and SAM), as well as the initial stages of flowering (FB1 – FB4). Particularly, attention was given to the four genes identified as homologous to those in *A. thaliana*: LOC101515142 (*CaMED16b*), LOC101489432 (*CaELF3a*), LOC101499101 (*BBX24-like*) and LOC101507442 (*VRN1-like*)*.* The TPM values, calculated from the chickpea expression atlas [52], categorized these genes into three different clusters (Fig. 7 and Additional file 12).

The expression profile of *CaMED16b* indicates higher expression levels during the initial stages of flowering, peaking between the SAM and FB1, followed by a decrease in subsequent FB stages (FB2 – FB4). This indicates a potential role for *CaMED16b* during the immediate pre-flowering period. In contrast, *CaELF3a* shows the highest expression levels in ML and SAM tissues, suggesting its involvement in upstream transcriptional regulation pathways preceding the onset of flowering. This suggests that *CaELF3a* may play a role in regulating processes leading up to flowering initiation. Finally, LOC101499101 and LOC101507442 exhibit a similar expression pattern, with the highest TPM levels observed at FB1 and consistent expression levels across all subsequent FB stages. This suggests their involvement during the flower development rather than the initiation of flowering. According to the in silico analysis, *CaMED16b* and *CaELF3a* exhibit interesting expression patterns consistent with an expected role in flowering time regulation, with expression in leaves and SAM preceding the transition from vegetative to reproductive stages. This observation aligns with previous reports identifying *CaELF3a* as a key regulator responsible for early inflorescence development and an early-flowering phenotype in chickpeas [98]*.* Interestingly, LOC101492009 (TIFY5A protein) and LOC101510831 (helicase-like transcription factor CHR28), both associated with the stress response GO Slim term, show similar expression patterns (Fig. 7). Basu et al. found that stress and defense-responsive genes as well as the ethylene signaling pathway genes were to be upregulated during inflorescence development in chickpeas [98].

Furthermore, other candidate genes, including some members of the ARF, ERF, and DOF families, exhibit co-expression with the four genes discussed previously **(**Table 4**)**. These transcription factor families play important roles in various fundamental processes in plants that could influence the phenotype observed for the pair of NILs [62–64, 99–101]. Specifically, *ARF2* and *ERF12* were described in *A. thaliana* with roles in flowering. *A. thaliana arf2* mutants exhibited pleiotropic development phenotypes, including delays in several processes related to plant aging such as initiation of flowering, rosette leaf senescence and floral organ abscission [102, 103]. *Arabidopsis EFR12* pleiotropically affects meristem identity, floral phyllotaxy and organ initiation and seems to be conserved among angiosperms [104]. Therefore, LOC101513952 (*CaARF2;* auxin response factor 2), with 14 variants detected between NILs and LOC101491273 (*CaERF12*; ethylene-responsive transcription factor 12), affected by four HHQ-I-C/E variants, are potential candidate genes that could play a role in the observed phenotypic differences.

## Conclusion

The development of the NIL pair in this study represents a valuable resource for advancing research on chickpea flowering time. This study offers a complementary approach to association analyses by phenotyping and resequencing the NILs, enabling the identification of candidate gene variants that could have both major and minor effects on flowering time. While *CaELF3a* emerges as the most prominent candidate gene, our study also uncovered other targets for the first time in chickpea, including *CaMED16b* and LOC101499101 (*BBX24-like*), which are homologs to flowering-related genes in *A. thaliana*. This suggests their potential contribution in modeling this trait. Furthermore, ERF and ARF family members potentially associated with flowering time were also detected. The in silico expression characterization and genetic variability analysis carried out in this study for these loci could contribute to the development of specific markers for chickpea breeding programs. This study lays the foundation for future research on this plant material. Subsequent studies, including analysis of the F2 progeny resulting from the NIL cross and expression analysis, hold the potential to unveil new insights into the intricate mechanisms governing flowering time in chickpea.

### Supplementary Information

Additional file 1. Variants detected between the chickpea near-isogenic lines pair differing in flowering time.

Additional file 2. GO terms enriched in the dataset of 306 flowering-related genes in *Arabidopsis thaliana* obtained from the FLOR-ID with the corresponding GO Slim term.

Additional file 3. Homozygous variants located in genes (HHQ-I variants).

Additional file 4. Description of the non-protein-coding genes affected by HHQ-I variants.

Additional file 5. Functional annotation of the protein-coding genes with variants in exon or CDS (HHQ-I-C/E variants) and enrichment analysis calculated via Fisher’s exact test comparing the functional annotations of the protein-coding genes with HHQ-I-C/E variants against the whole chickpea genome annotation.

Additional file 6. List of 146 protein-coding genes with GO Slim terms enriched in the 306 genes FLOR-ID *Arabidopsis thaliana* dataset.

Additional file 7. Diagrams of the location of HHQ-I-C/E variations on (a) LOC101515142, (b) LOC101489432 (*CaELF3a*), (c) LOC101499101 and (d) LOC101507442.

Additional file 8. Conservation of the LOC101515142 haplotypes detected in the NIL pair across 3,171 cultivated *Cicer arietinum* accessions.

Additional file 9. DTF and genotypic data (SNPs present in LOC101515142 haplotype, LOC101499101, and LOC101507442) for 3,171 cultivated chickpea accessions available in the public repository CicerSeq.

Additional file 10. DTF distribution for cultivated chickpea accessions according to their genotype.

Additional file 11. Protein–protein interaction network using STRING chickpea database for (a) LOC101515142 and (b) LOC101499101.

Additional file 12. TPM data for 132 transcript ID and their corresponding matched gene ID.

Additional file 13. Observed heterozygosity calculated in cultivated chickpea lines according to the heterozygous SNPs data reported by Varshney et al. (2021).

Additional file 14. BLASTp *Medicago truncatula MED16A* and *MED16B* against *Cicer arietinum* RefSeq_Protein database.

## Data Availability

The dataset generated and analyzed during the current study are available in the European Variation Archive (EVA) at EMBL-EBI under accession number PRJEB73790, https://urldefense.com/v3/__https://www.ebi.ac.uk/eva/?eva-study=PRJEB73790__;!!D9dNQwwGXtA!UdDPickLBLigeaKK1uTr009AH7xm-vup0ndN_fMEwYr8Ay_ik2ooSI7MDZSsXYi4d24v4nT4KWYkv8qN57t0o20q$. The custom scripts used for the analysis of the data during the current study are available in the GitHub repository https://github.com/AGR114molecularBreeding/chickpea.
