# Supplementary material for: Phenotypic and genetic characterization of a near-isogenic line pair: insights into flowering time in chickpea
Source: BMC Plant Biol. 2024 Jul 25;24:709. doi: 10.1186/s12870-024-05411-y (PMC11270784; doi:10.1186/s12870-024-05411-y)
Supplement: Supplementary file 7 — Additional file 7. Diagrams of the location of HHQ-I-C/E variations on (a) LOC101515142, (b) LOC101489432 (CaELF3a), (c) LOC101499101 and (d) LOC101507442. [file 12870_2024_5411_MOESM7_ESM.pdf]

a

LOC101515142

Mediator of RNA polymerase II transcription subunit 16-like

(Ca1: 2,285,592 – 2,298,911, complement)

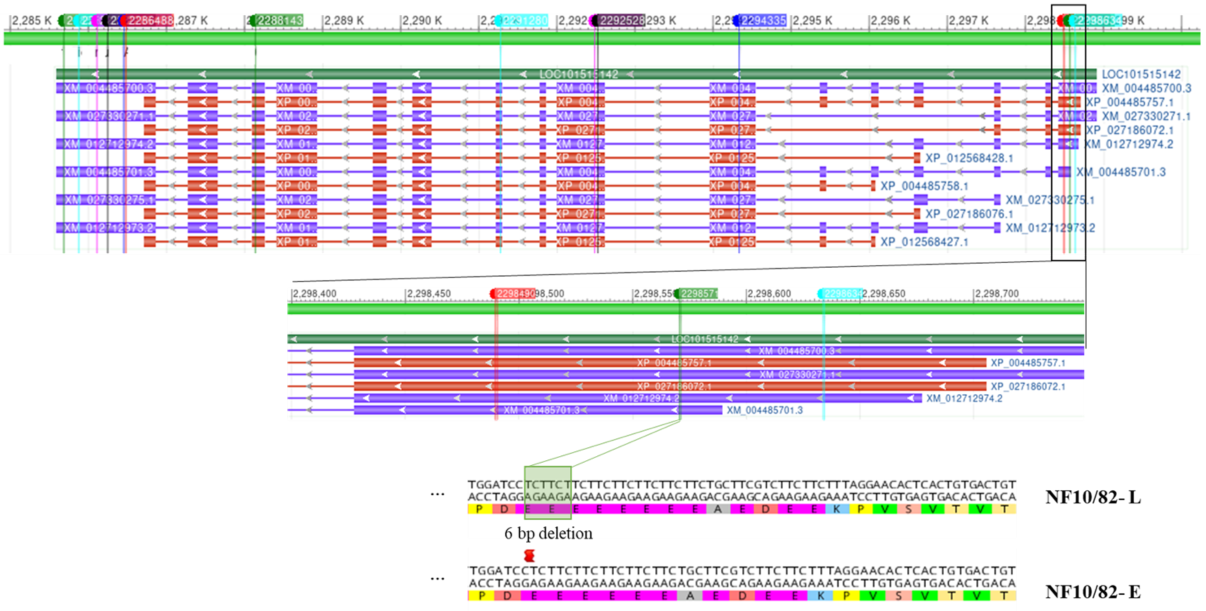

b

LOC101489432

protein EARLY FLOWERING 3a

(Ca5: 36,011,384 – 36,016,600, complement)

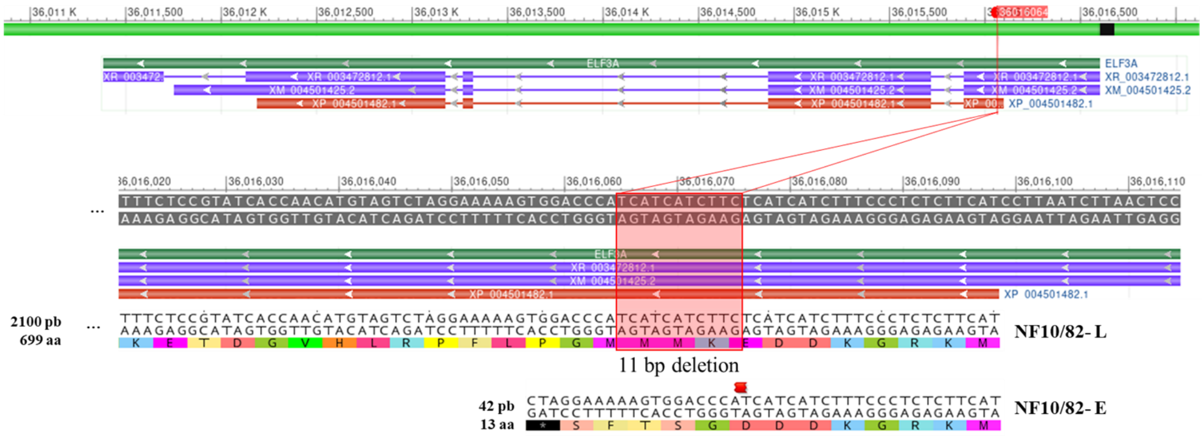

c

LOC101499101
B-box zinc finger protein 24
(Ca6: 57,549,424 – 57,552,323, complement)

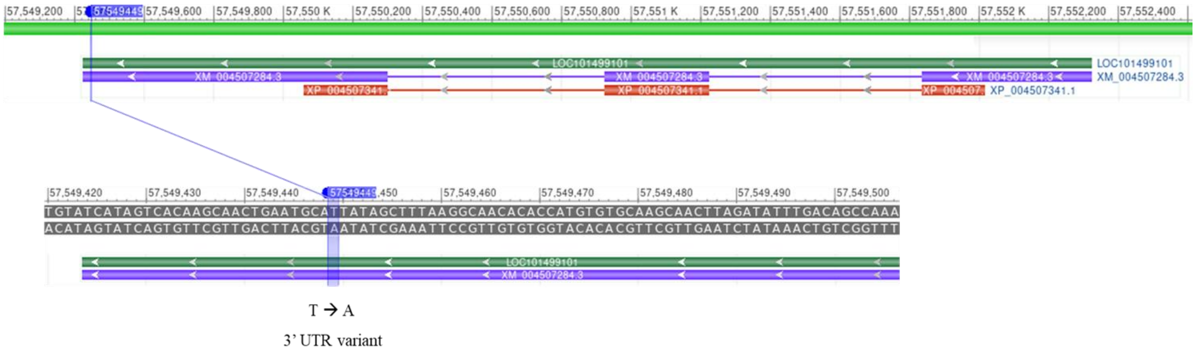

d

LOC101507442
B3 domain-containing transcription factor VRN1-like
(Ca6: 57,717,926 – 57,721,229)

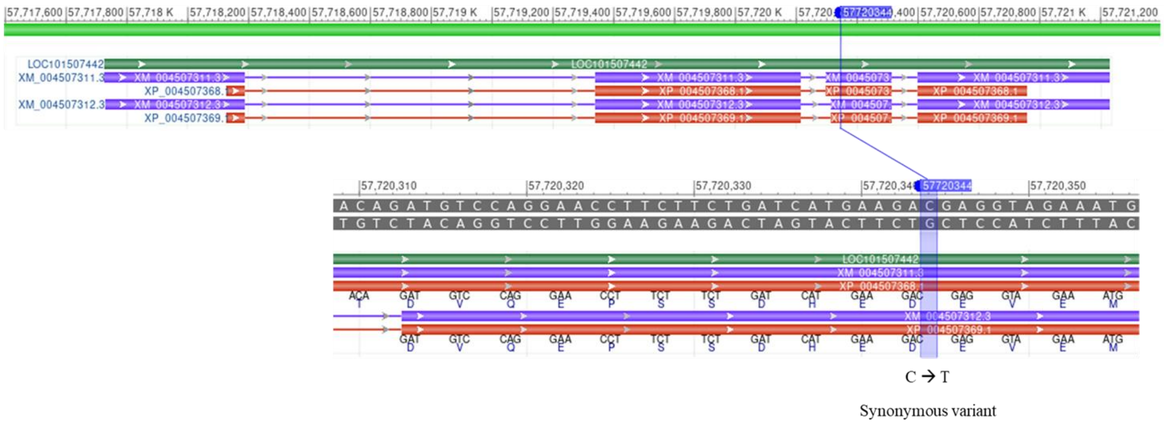

Additional file 7. Fig. S1 Diagrams of the location of HHQ-I-C/E variations on (**a**) LOC101515142, (**b**) LOC101489432 (*CaELF3a*), (**c**) LOC101499101 and (**d**) LOC101507442
