# Supplementary material for: Phenotypic and genetic characterization of a near-isogenic line pair: insights into flowering time in chickpea
Source: BMC Plant Biol. 2024 Jul 25;24:709. doi: 10.1186/s12870-024-05411-y (PMC11270784; doi:10.1186/s12870-024-05411-y)
Supplement: Supplementary file 10 — Additional file 10. DTF distribution for cultivated chickpea accessions according to their genotype. [file 12870_2024_5411_MOESM10_ESM.pdf]

Additional file 10. Table S1 DTF (mean ± SD) for chickpea accessions at 6 locations acquired from the public repository CicerSeq (Varshney et al. 2021).

Haplotype		N	ICARDA 14/15	ICARDA 15/16	ICRISAT 14/15	ICRISAT 15/16	IIPR 15/16	JAU 14/16	JAU 15/16	RAKCA 14/15	RAKCA 15/16	RARI 14/15	RARI 15/16
LOC 101515142 *MED16*-like	Hap1	2219	90.4 ± 5.90	64.2 ± 8.43	56.8 ± 7.87	66.5 ± 16.0	71.3 ± 6.67	63.6 ± 8.30	66.0 ± 8.96	79.0 ± 4.71	70.8 ± 5.97	85.1 ± 8.63	82.5 ± 6.37
	Hap2	749	90.7 ± 6.13	64.5 ± 8.36	57.5 ± 8.50	67.0 ± 17.4	71.3 ± 6.86	64.0 ± 8.54	66.1 ± 9.38	79.5 ± 4.60	71.2 ± 5.41	88.2 ± 9.34	82.4 ± 6.17
	t-test		ns	ns	ns	ns	ns	ns	ns	*	ns	***	ns
LOC 101499101 B-box zinc finger protein 24	T	2609	90.4 ± 5.94	64.1 ± 8.24	56.8 ± 7.79	66.0 ± 16.0	71.2 ± 6.69	63.5 ± 8.32	65.8 ± 9.03	79.1 ± 4.63	70.8 ± 5.77	85.8 ± 8.89	82.5 ± 6.23
	A	299	91.3 ± 5.94	66.8 ± 9.21	62.3 ± 7.33	76.7 ± 15.8	73.6 ± 6.26	68.2 ± 7.38	70.0 ± 7.84	80.2± 4.85	72.1± 6.35	90.0± 8.75	82.4± 6.85
	t-test		*	***	***	***	***	***	***	***	**	***	ns
LOC 101507442 *VRN1*-like	C	2654	90.5 ± 5.91	64.2 ± 8.27	57.3 ± 7.98	66.9 ± 16.2	71.3 ± 6.67	63.9 ± 8.24	66.2 ± 8.85	79.2 ± 4.58	70.9 ± 5.82	86.1 ± 8.92	82.5 ± 6.28
	T	275	90.8 ± 5.94	65.9 ± 9.88	57.0 ± 8.00	67.7 ± 17.3	72.5 ± 7.06	64.0 ± 9.46	66.2 ± 10.7	79.0 ± 5.80	71.7 ± 6.57	85.8 ± 8.99	82.5 ± 6.46
	t-test		ns	*	ns	ns	*	ns	ns	ns	ns	ns	ns
Mean ± SD			90.5 ± 5.96	64.3 ± 8.39	57.2 ± 7.98	66.9 ± 16.24	71.4 ± 6.70	63.9 ± 8.34	66.2 ± 8.99	79.2 ± 4.66	70.9 ± 5.83	86.1 ± 8.95	82.5 ± 6.33

Significant difference Student's t-test (ns: non-significant, *0.01 < $P \leq 0.05$, **0.001 < $P \leq 0.01$, ***$P \leq 0.001$).

a LOC101515142 haplotypes

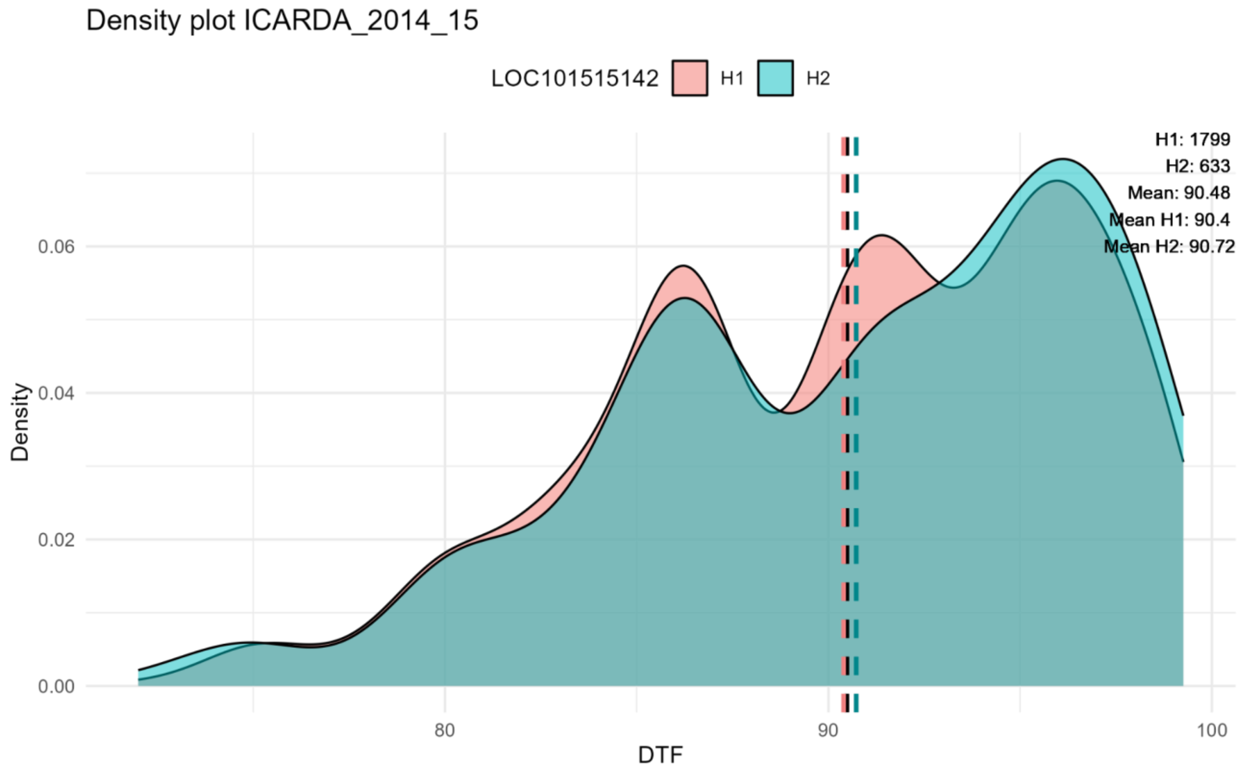

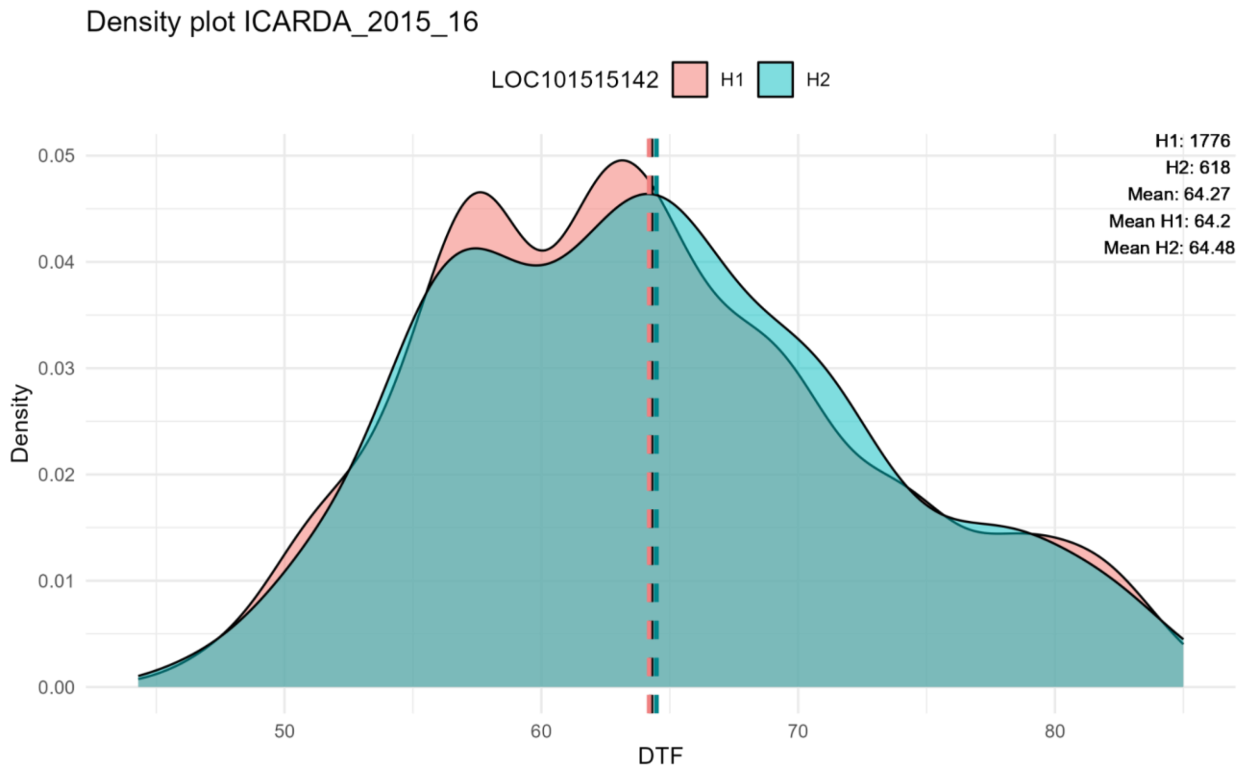

Density plot ICARDA_2015_16
LOC101515142
H1
H2
H1: 1776
H2: 618
Mean: 64.27
Mean H1: 64.2
Mean H2: 64.48
Density
0.05
0.04
0.03
0.02
0.01
0.00
50
60
70
80
DTF

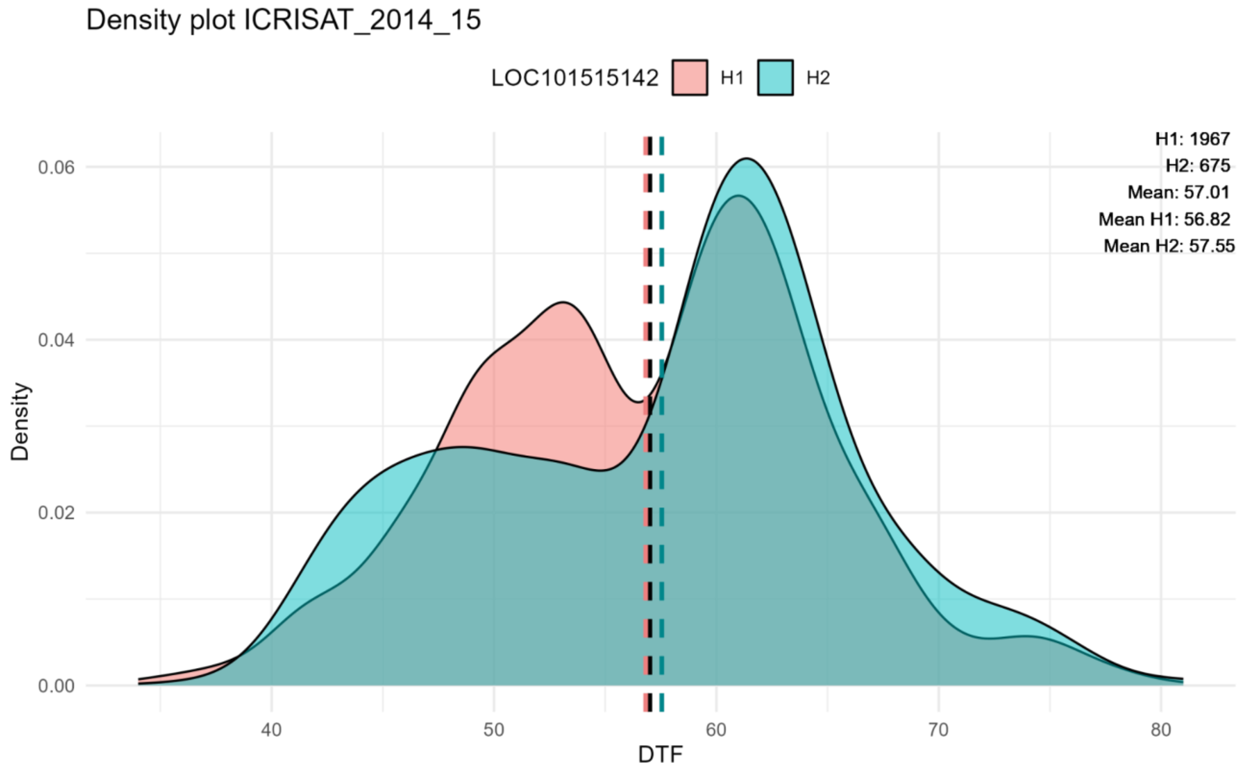

Density plot ICRISAT_2014_15
LOC101515142
H1
H2
H1: 1967
H2: 675
Mean: 57.01
Mean H1: 56.82
Mean H2: 57.55
Density
0.06
0.04
0.02
0.00
40
50
60
70
80
DTF

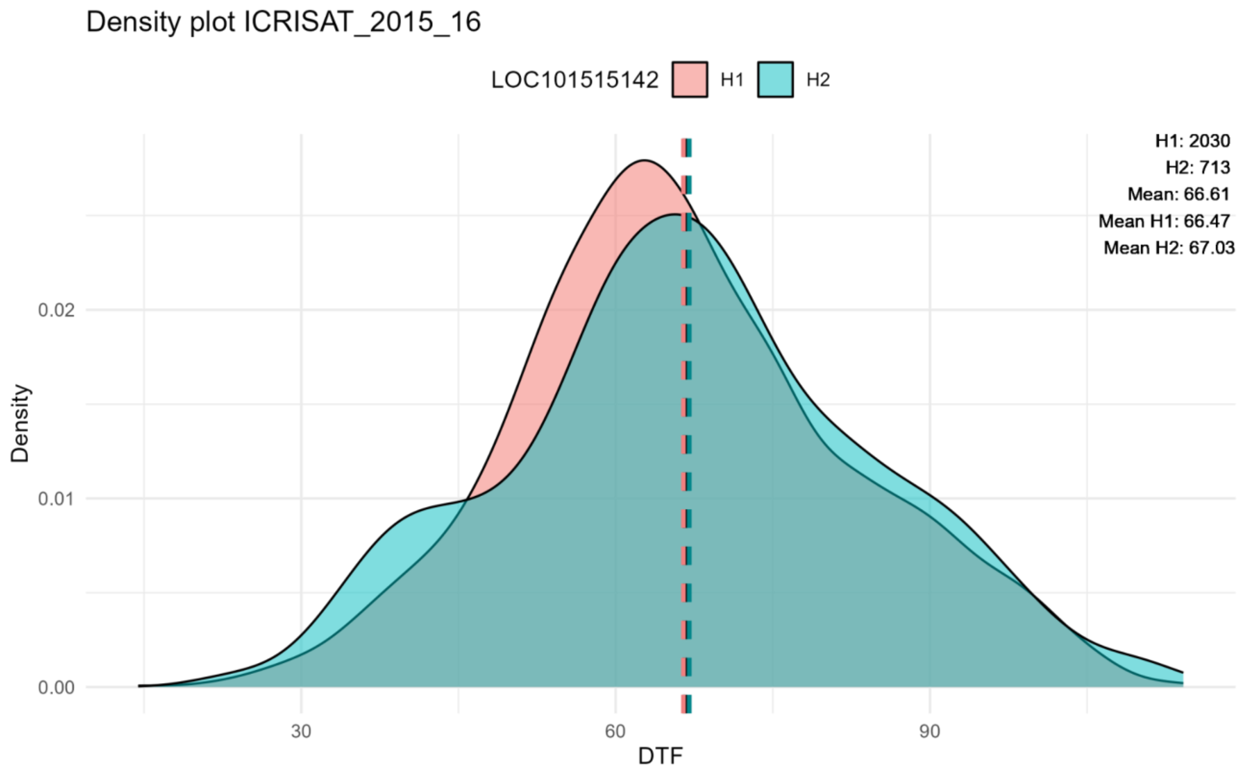

Density plot ICRISAT_2015_16
LOC101515142
H1
H2
H1: 2030
H2: 713
Mean: 66.61
Mean H1: 66.47
Mean H2: 67.03
Density
0.00
0.01
0.02
30
60
90
DTF

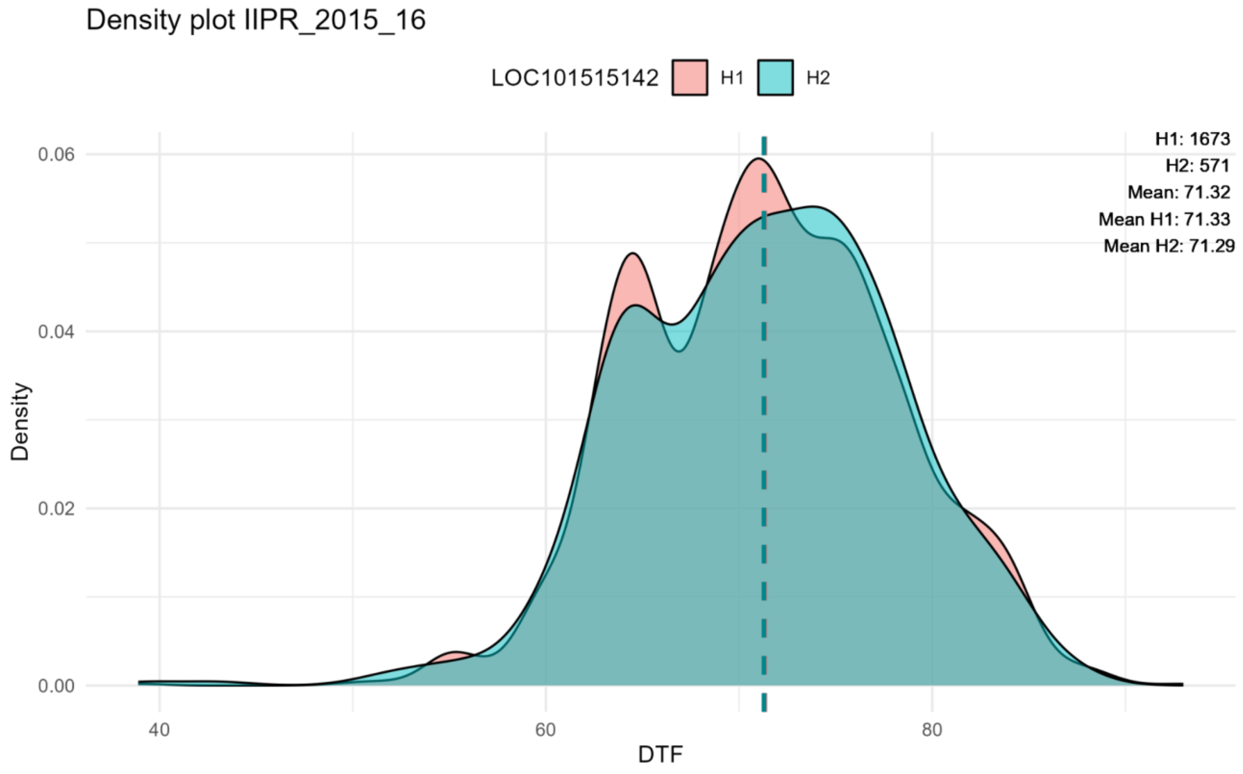

Density plot IIPR_2015_16
LOC101515142
H1
H2
H1: 1673
H2: 571
Mean: 71.32
Mean H1: 71.33
Mean H2: 71.29
Density
0.00
0.02
0.04
0.06
40
60
80
DTF

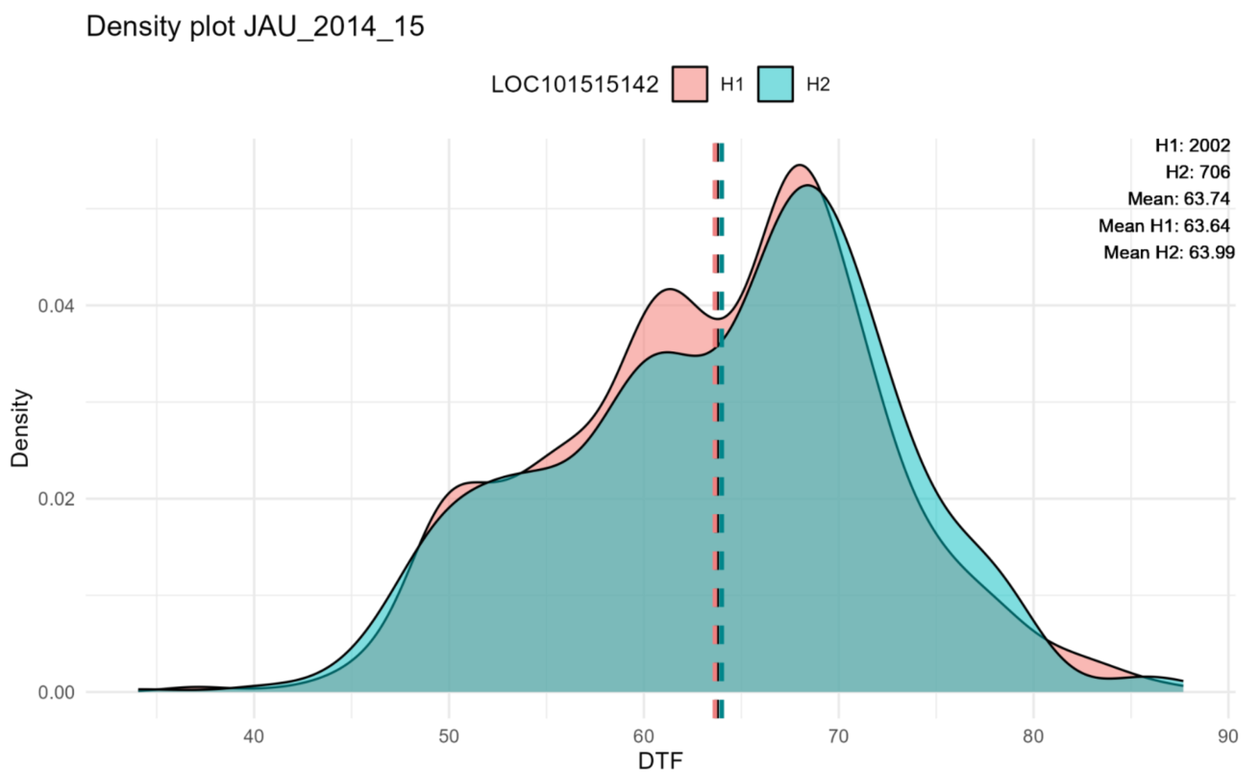
Density plot JAU_2014_15
LOC101515142
H1
H2
H1: 2002
H2: 706
Mean: 63.74
Mean H1: 63.64
Mean H2: 63.99
Density
0.00
0.02
0.04
40
50
60
70
80
90
DTF

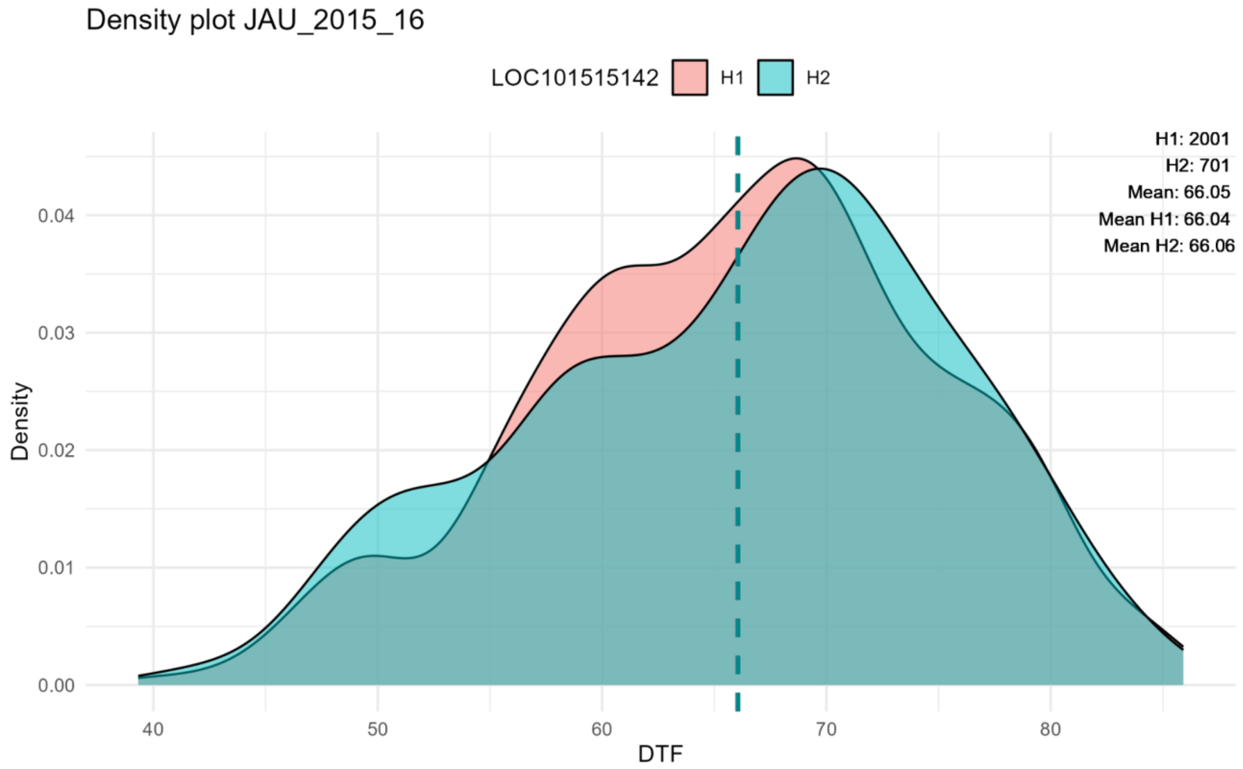
Density plot JAU_2015_16
LOC101515142
H1
H2
H1: 2001
H2: 701
Mean: 66.05
Mean H1: 66.04
Mean H2: 66.06
Density
0.00
0.01
0.02
0.03
0.04
40
50
60
70
80
DTF

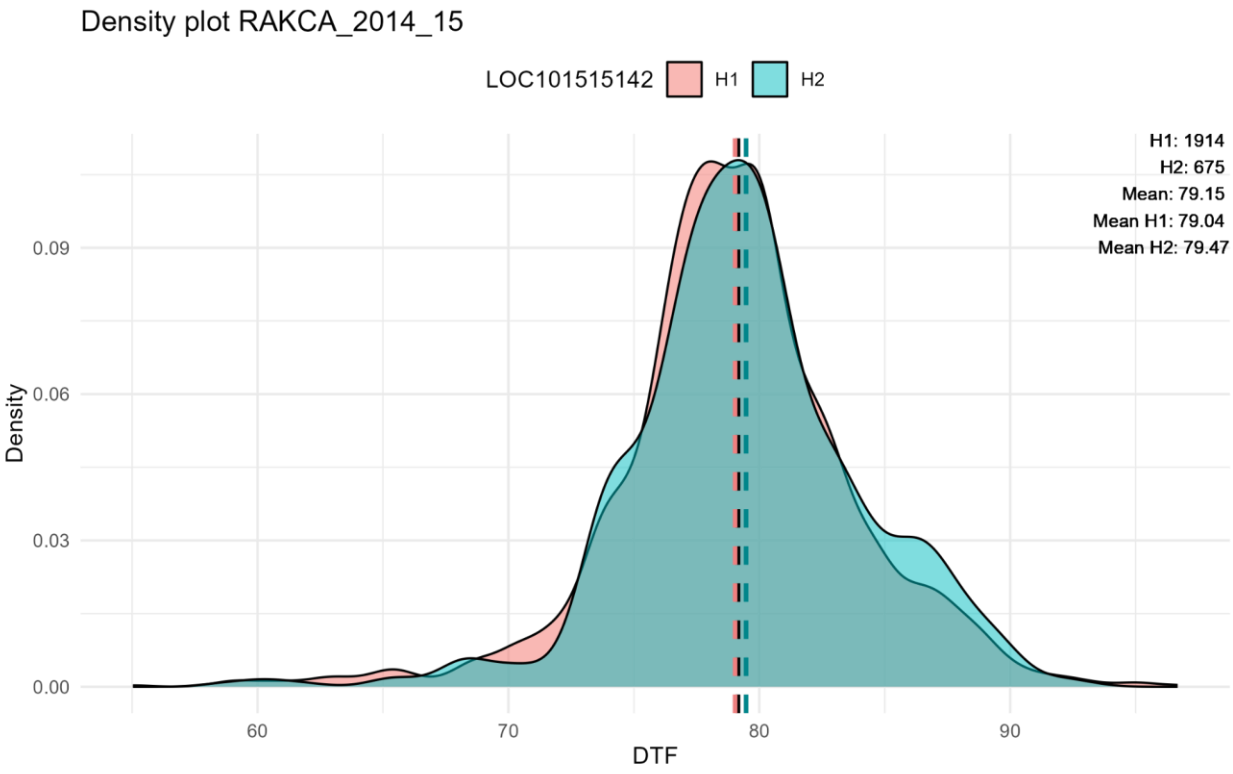
Density plot RAKCA_2014_15
LOC101515142
H1
H2
H1: 1914
H2: 675
Mean: 79.15
Mean H1: 79.04
Mean H2: 79.47
0.09
0.06
0.03
0.00
Density
60
70
80
90
DTF

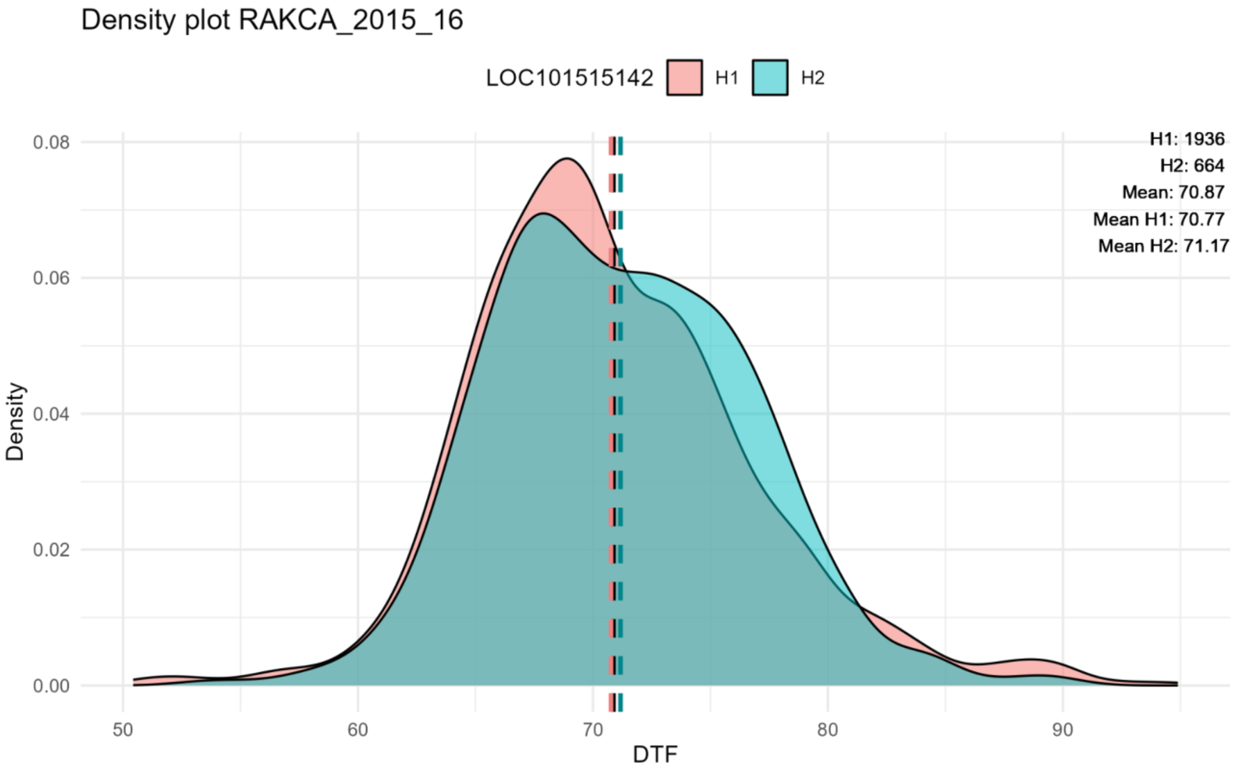
Density plot RAKCA_2015_16
LOC101515142
H1
H2
H1: 1936
H2: 664
Mean: 70.87
Mean H1: 70.77
Mean H2: 71.17
0.08
0.06
0.04
0.02
0.00
Density
50
60
70
80
90
DTF

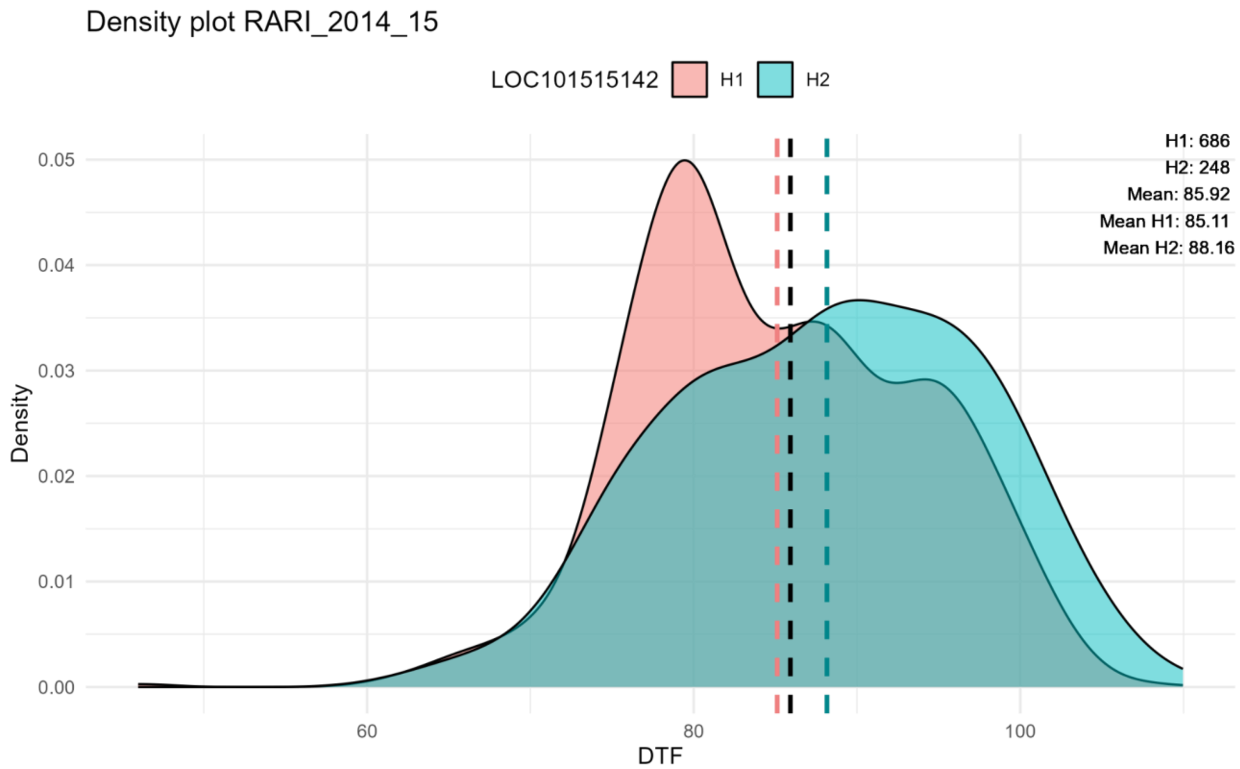
Density plot RARI_2014_15
LOC101515142
H1
H2
H1: 686
H2: 248
Mean: 85.92
Mean H1: 85.11
Mean H2: 88.16
Density
0.05
0.04
0.03
0.02
0.01
0.00
60
80
100
DTF

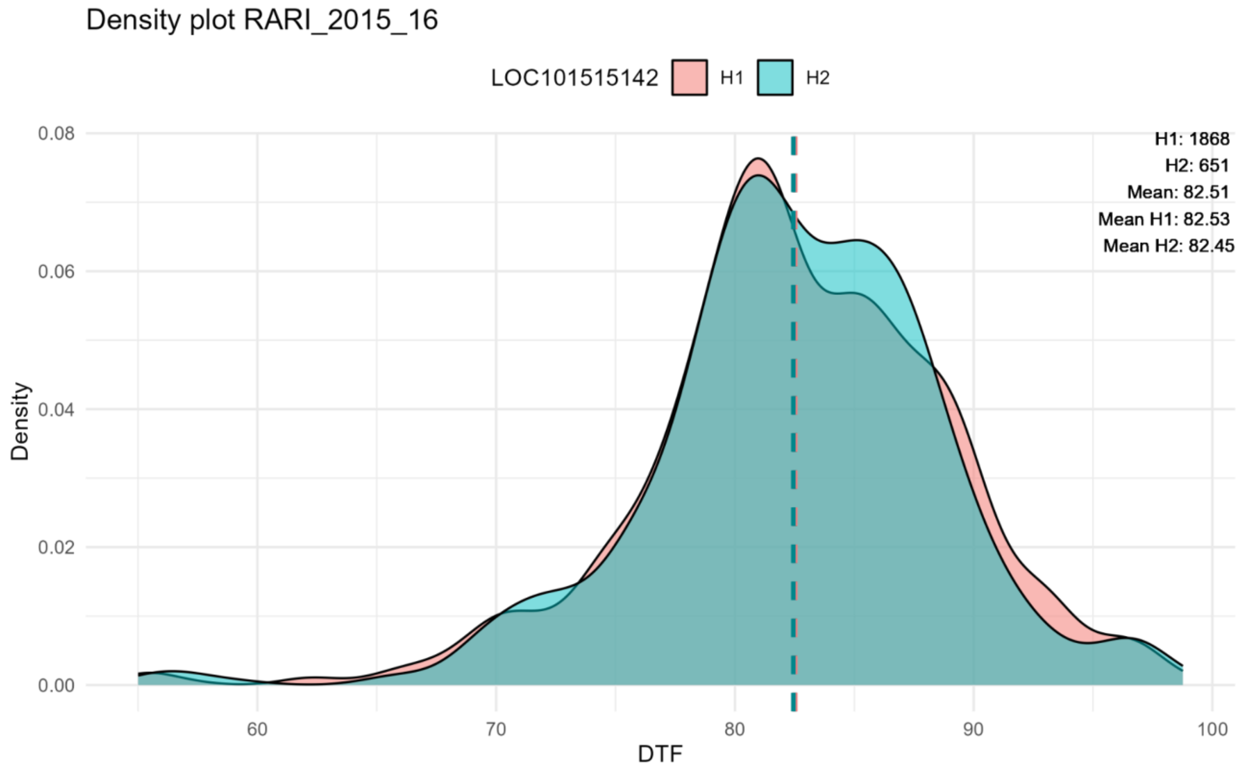
Density plot RARI_2015_16
LOC101515142
H1
H2
H1: 1868
H2: 651
Mean: 82.51
Mean H1: 82.53
Mean H2: 82.45
Density
0.08
0.06
0.04
0.02
0.00
60
70
80
90
100
DTF

b LOC101499101 SNP (Ca6: 57,549,449)

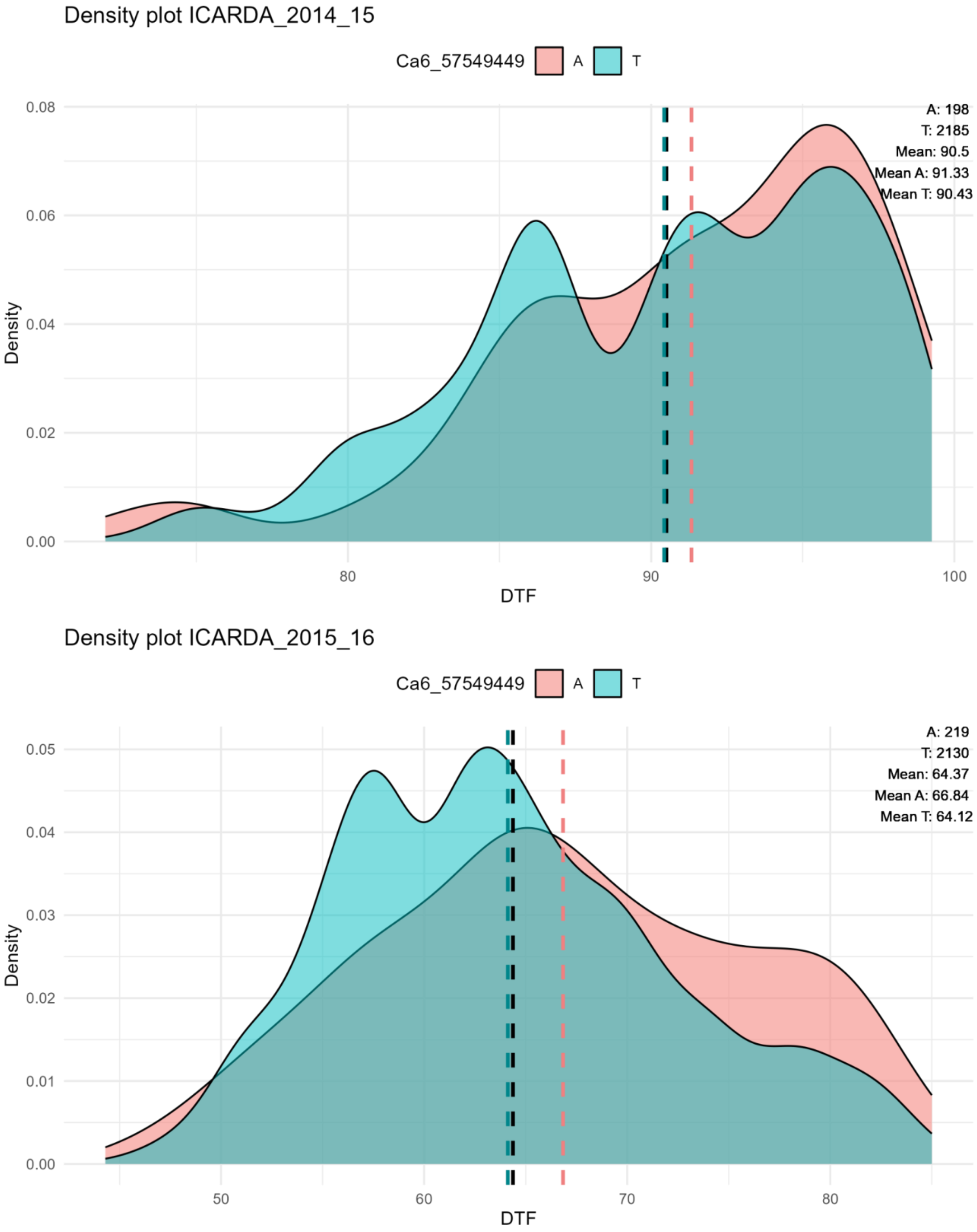

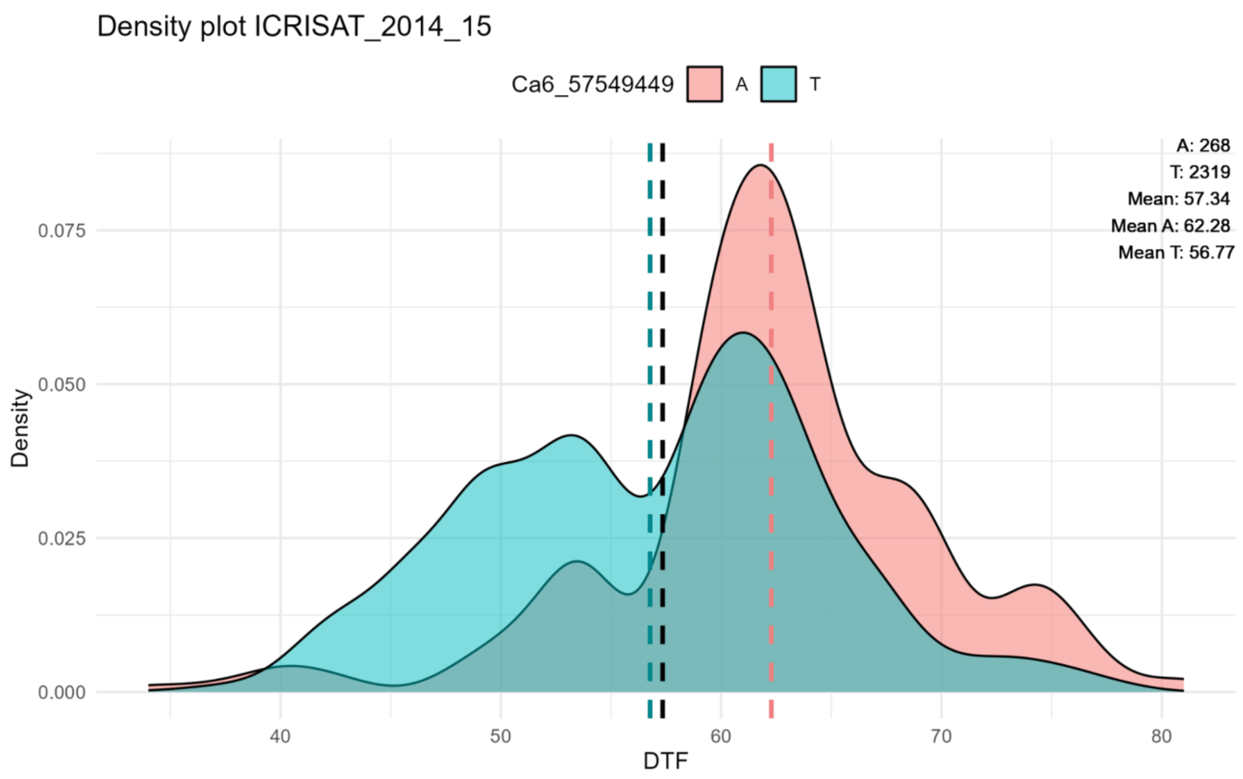
Density plot ICRISAT_2014_15
Ca6_57549449
A
T
A: 268
T: 2319
Mean: 57.34
Mean A: 62.28
Mean T: 56.77
Density
0.000
0.025
0.050
0.075
40
50
60
70
80
DTF

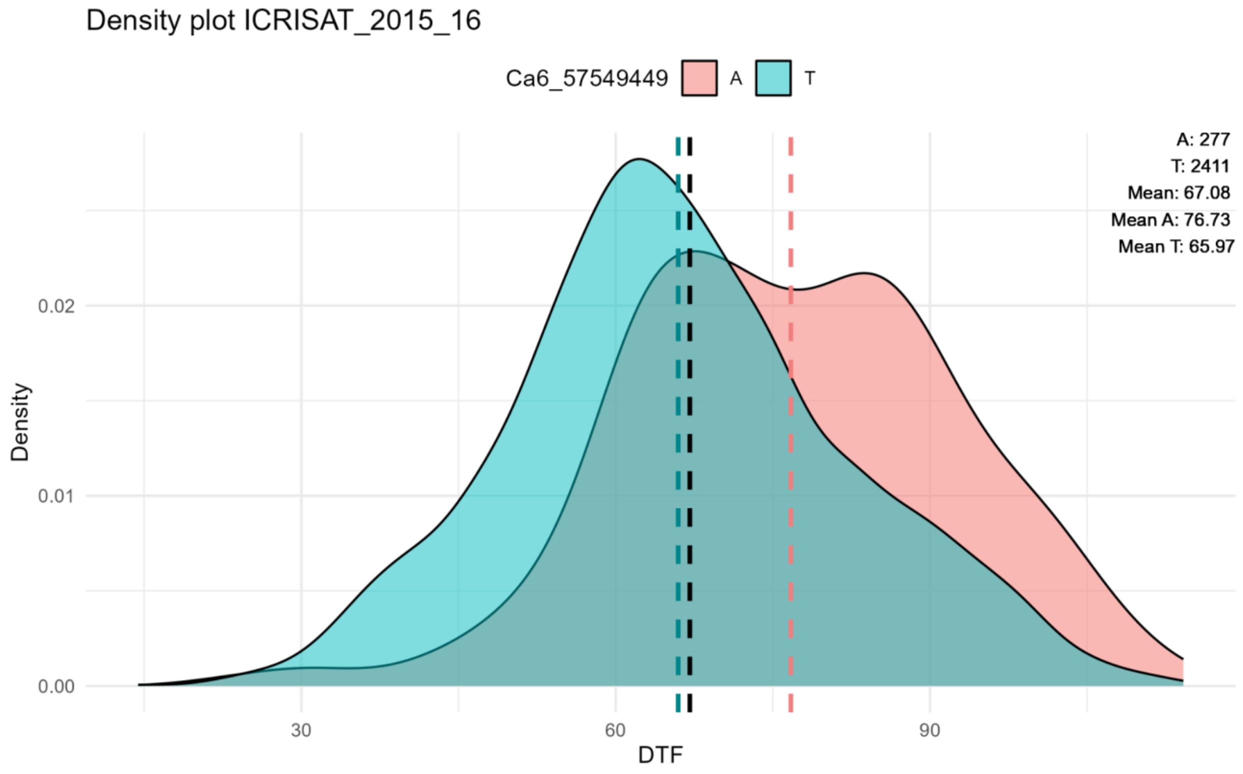
Density plot ICRISAT_2015_16
Ca6_57549449
A
T
A: 277
T: 2411
Mean: 67.08
Mean A: 76.73
Mean T: 65.97
Density
0.00
0.01
0.02
30
60
90
DTF

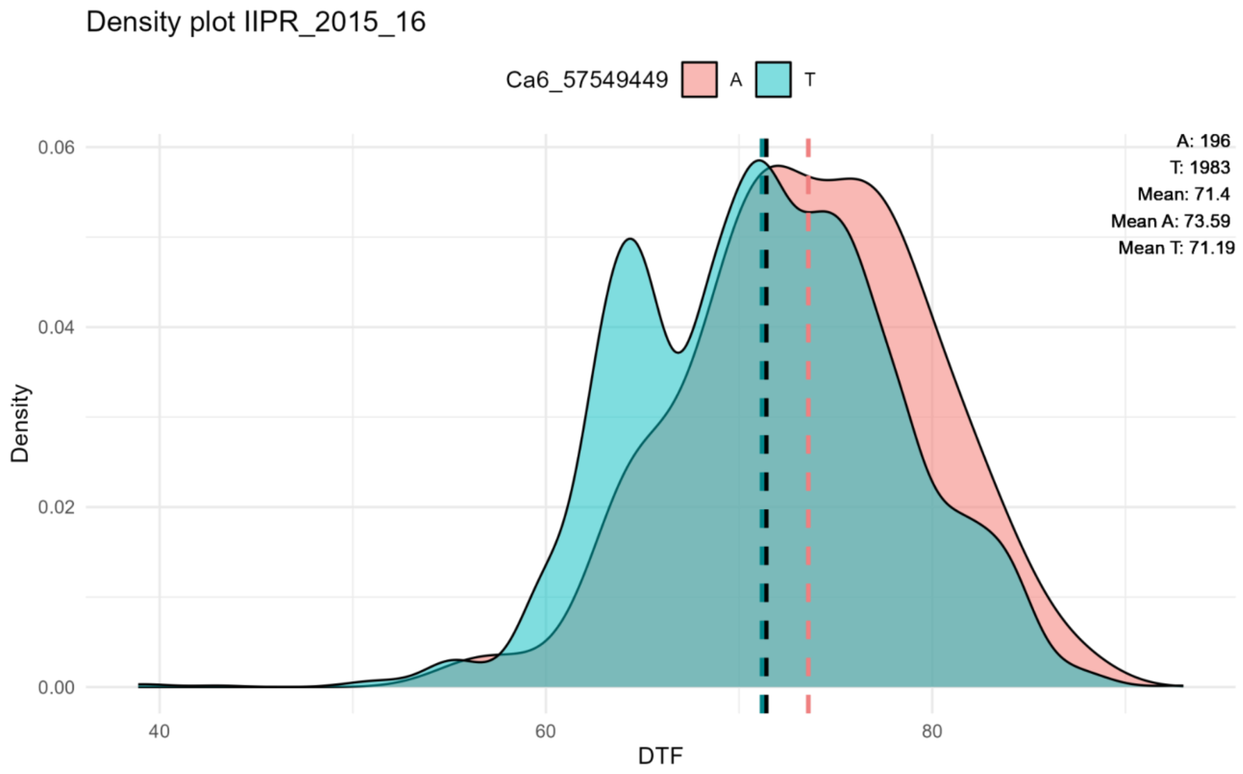
Density plot IIPR_2015_16
Ca6_57549449
A
T
A: 196
T: 1983
Mean: 71.4
Mean A: 73.59
Mean T: 71.19
Density
0.06
0.04
0.02
0.00
40
60
80
DTF

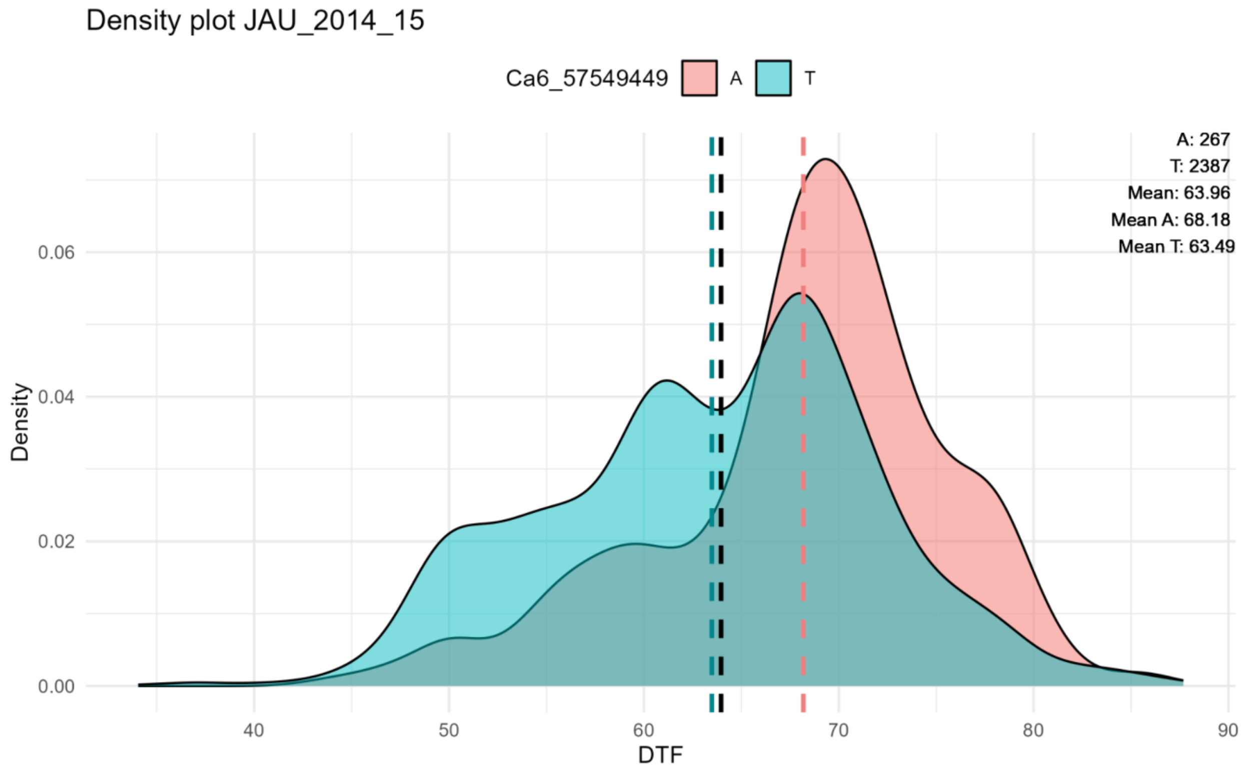
Density plot JAU_2014_15
Ca6_57549449
A
T
A: 267
T: 2387
Mean: 63.96
Mean A: 68.18
Mean T: 63.49
Density
0.06
0.04
0.02
0.00
40
50
60
70
80
90
DTF

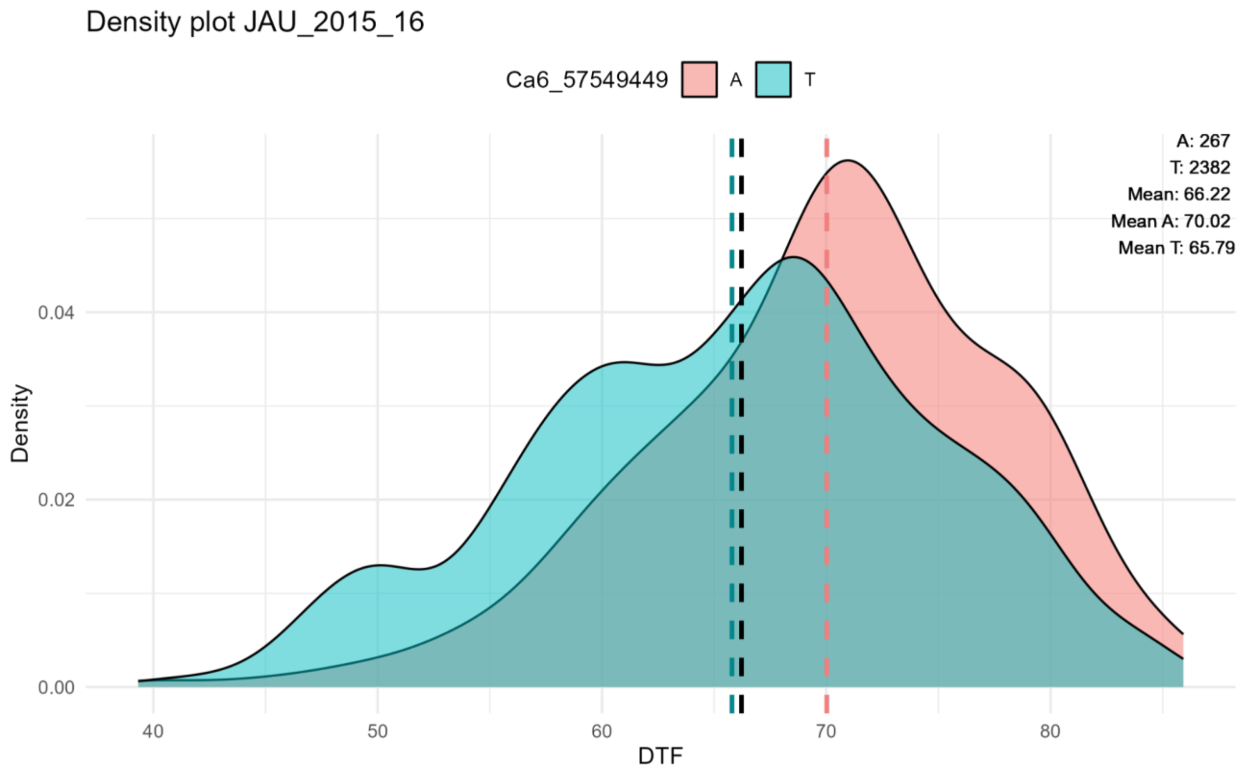

Density plot JAU_2015_16
Ca6_57549449
A
T
A: 267
T: 2382
Mean: 66.22
Mean A: 70.02
Mean T: 65.79
Density
0.00
0.02
0.04
40
50
60
70
80
DTF

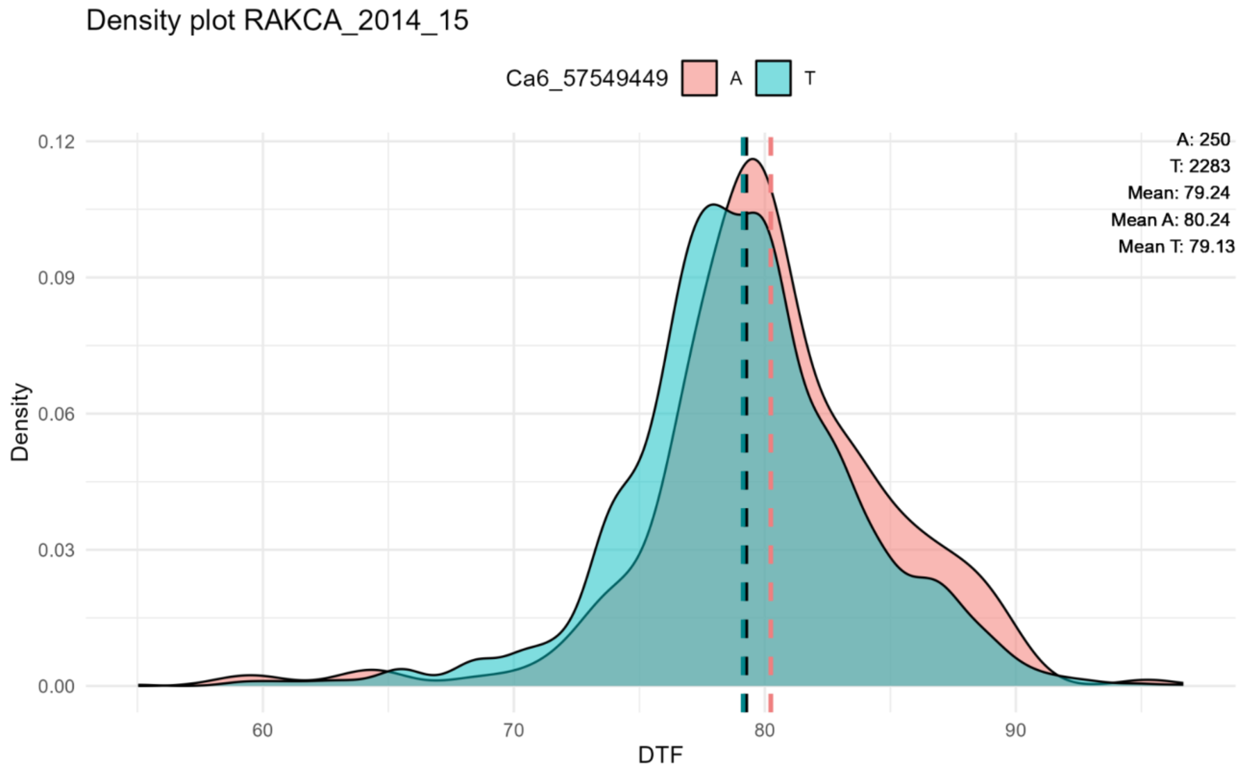

Density plot RAKCA_2014_15
Ca6_57549449
A
T
A: 250
T: 2283
Mean: 79.24
Mean A: 80.24
Mean T: 79.13
Density
0.00
0.03
0.06
0.09
0.12
60
70
80
90
DTF

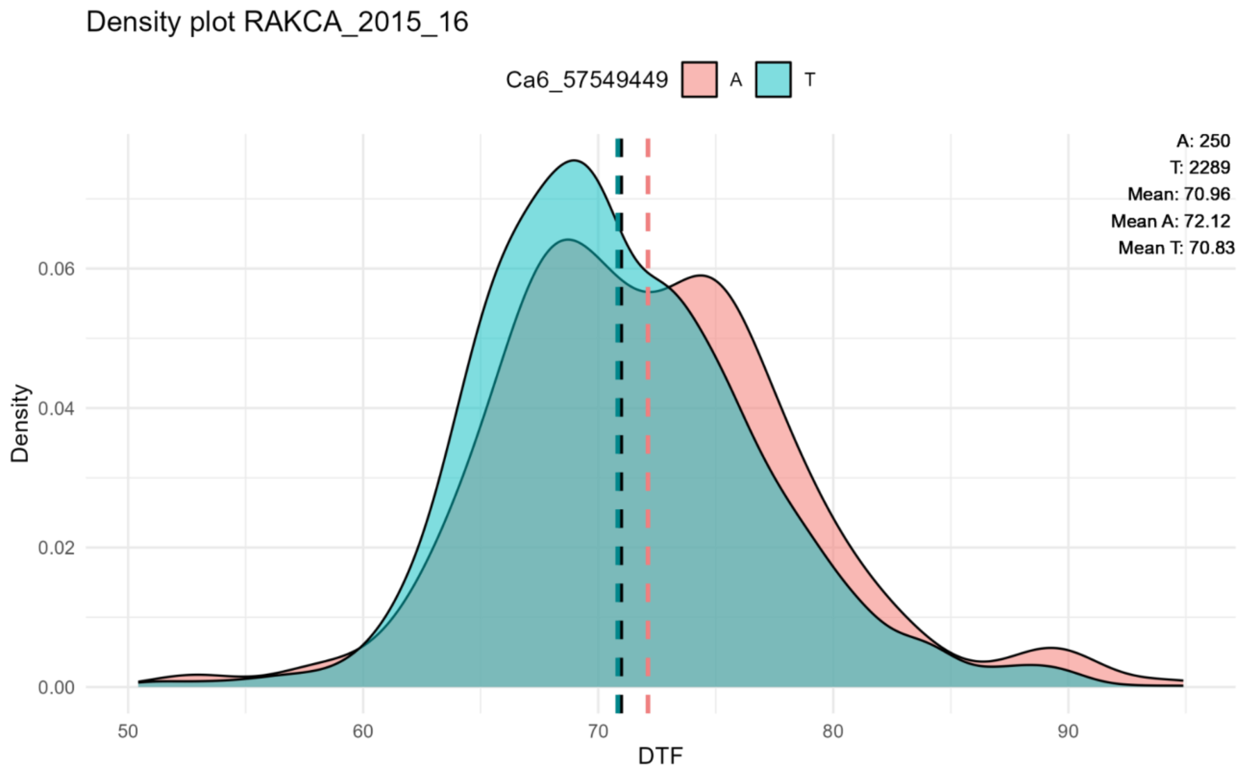

Density plot RAKCA_2015_16
Ca6_57549449
A
T
A: 250
T: 2289
Mean: 70.96
Mean A: 72.12
Mean T: 70.83
Density
0.06
0.04
0.02
0.00
50
60
70
80
90
DTF

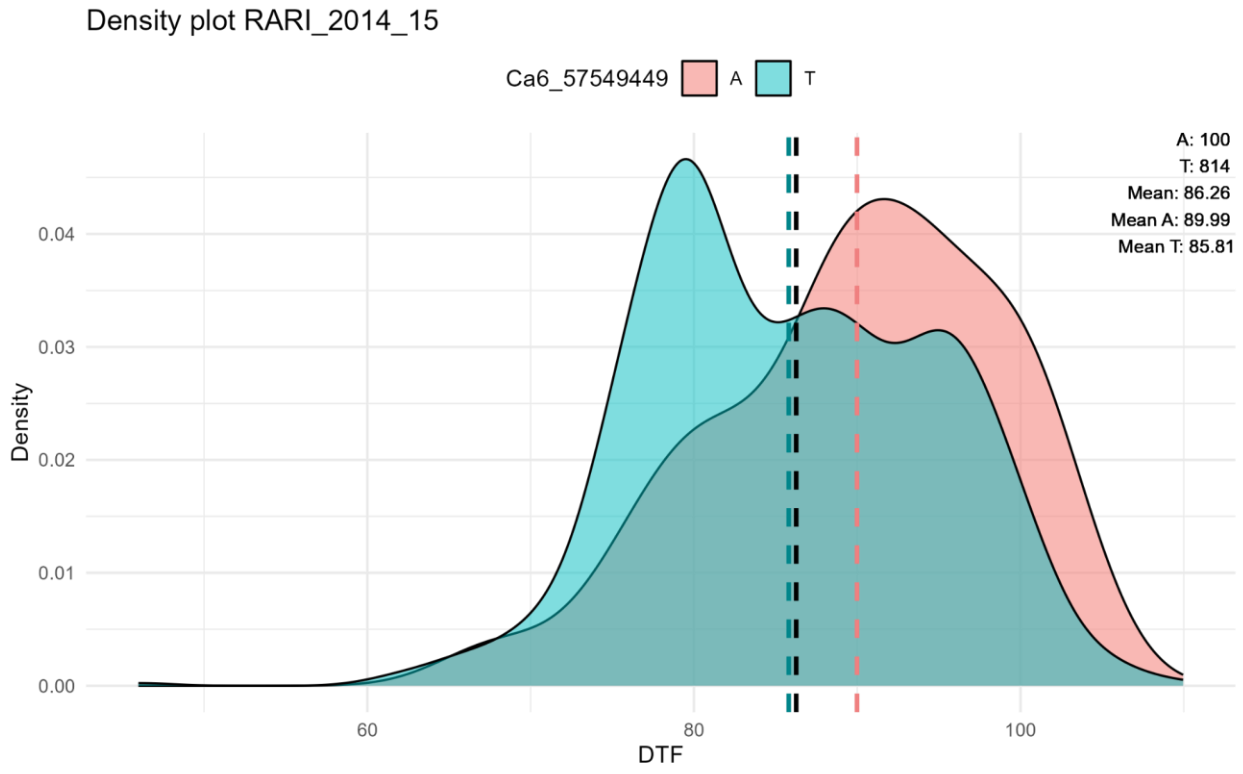

Density plot RARI_2014_15
Ca6_57549449
A
T
A: 100
T: 814
Mean: 86.26
Mean A: 89.99
Mean T: 85.81
Density
0.04
0.03
0.02
0.01
0.00
60
80
100
DTF

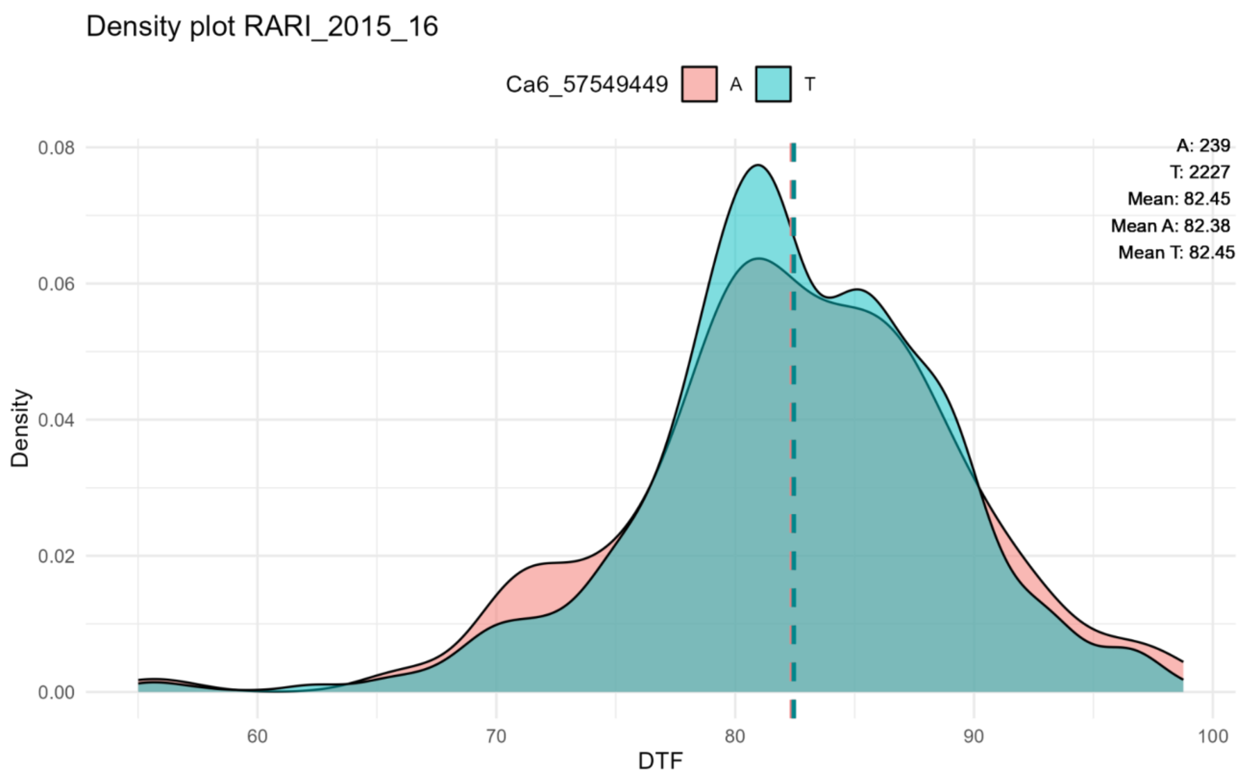

Density plot RARI_2015_16
Ca6_57549449
A
T
A: 239
T: 2227
Mean: 82.45
Mean A: 82.38
Mean T: 82.45
0.08
0.06
0.04
0.02
0.00
Density
60
70
80
90
100
DTF

c LOC101507442 SNP (Ca6: 57,720,344).

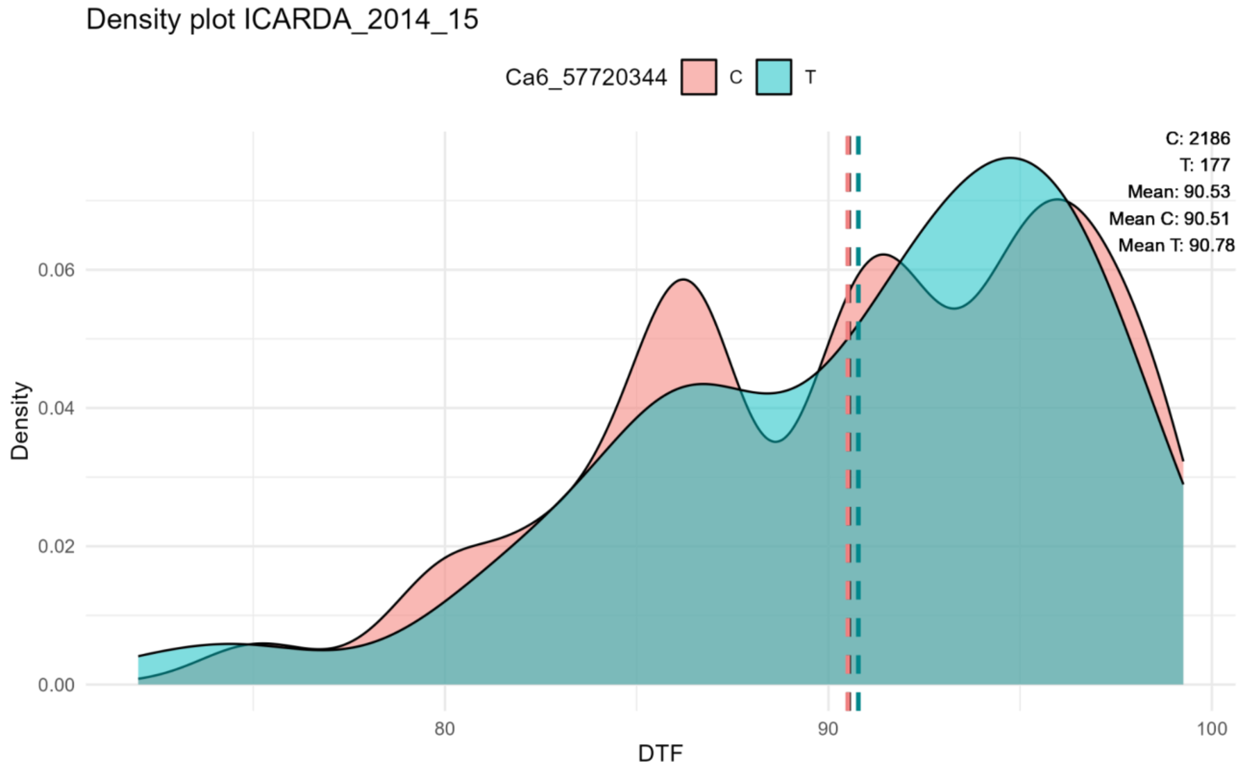

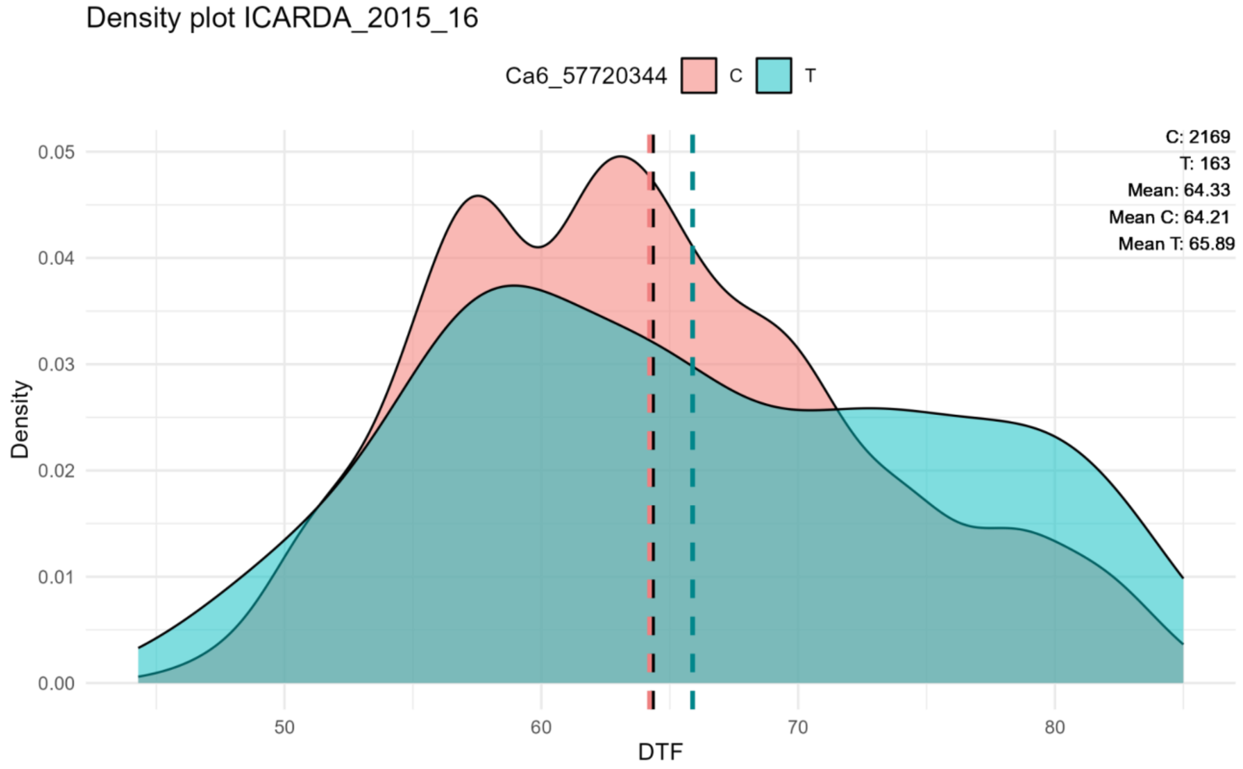

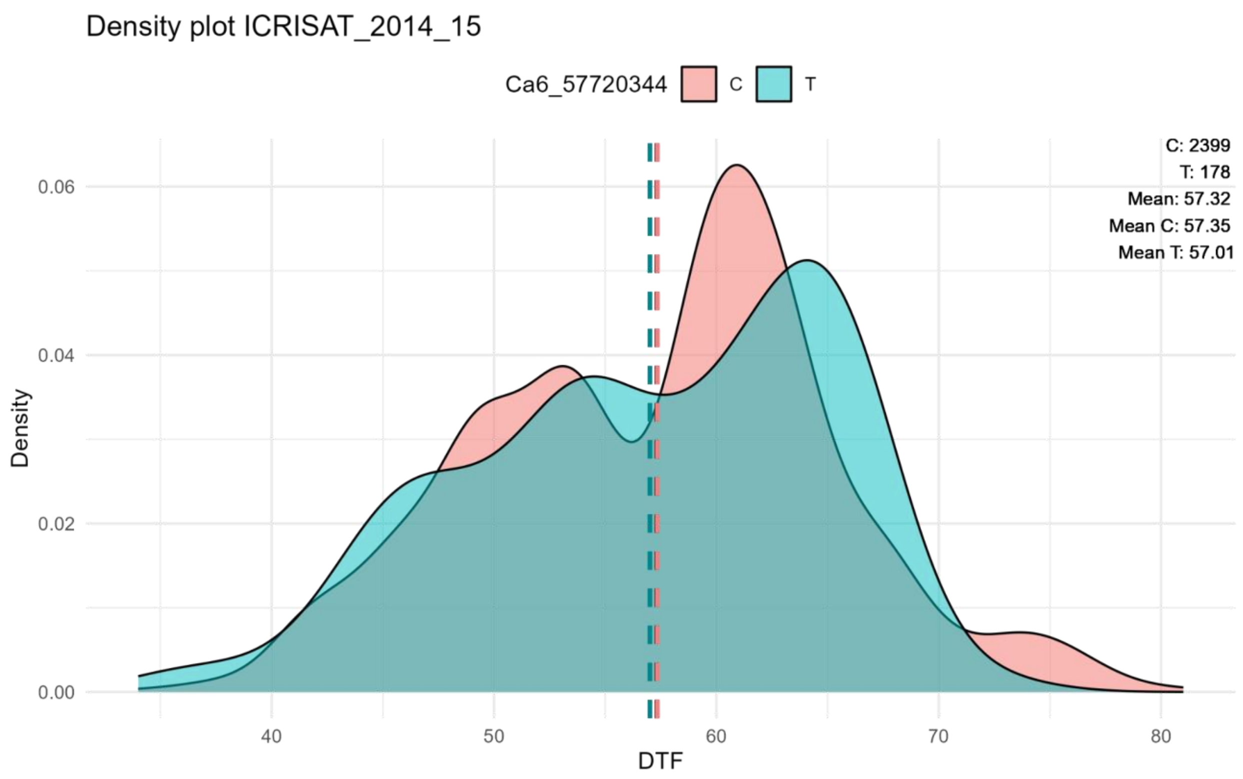
Density plot ICRISAT_2014_15
Ca6_57720344
C
T
C: 2399
T: 178
Mean: 57.32
Mean C: 57.35
Mean T: 57.01
Density
0.00
0.02
0.04
0.06
40
50
60
70
80
DTF

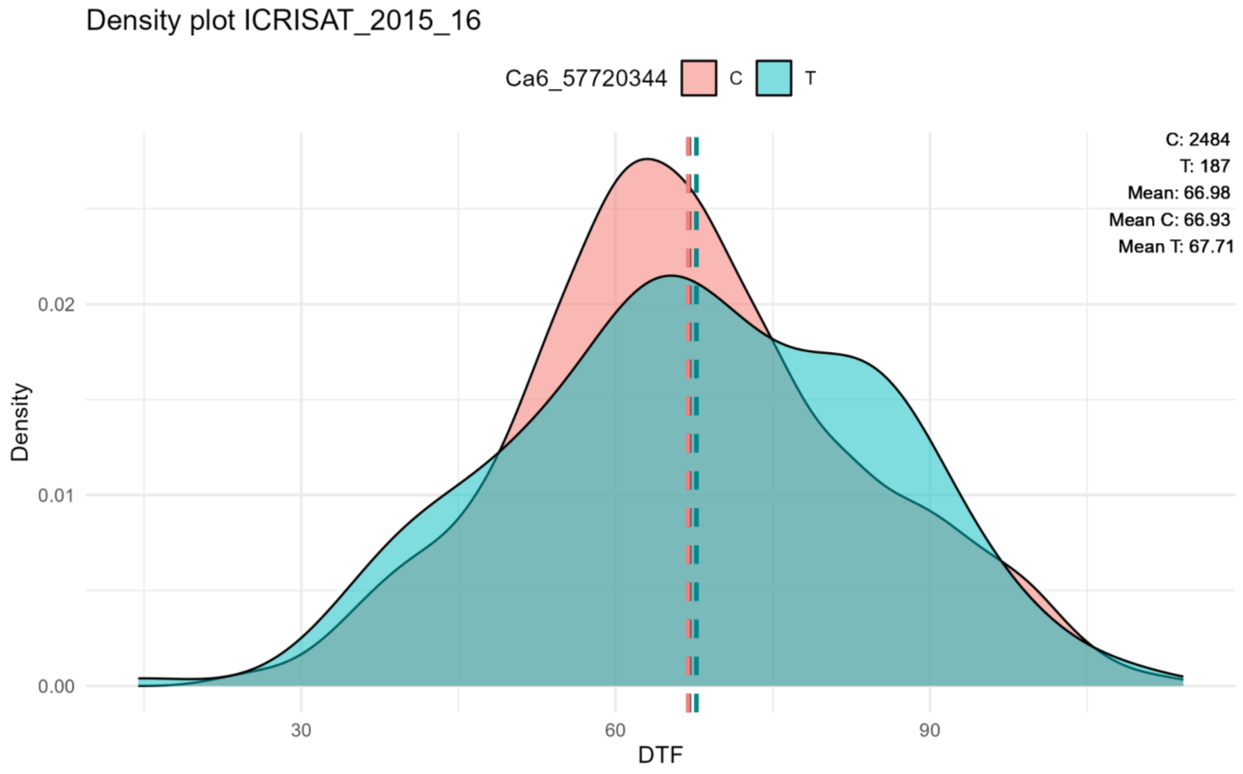
Density plot ICRISAT_2015_16
Ca6_57720344
C
T
C: 2484
T: 187
Mean: 66.98
Mean C: 66.93
Mean T: 67.71
Density
0.00
0.01
0.02
30
60
90
DTF

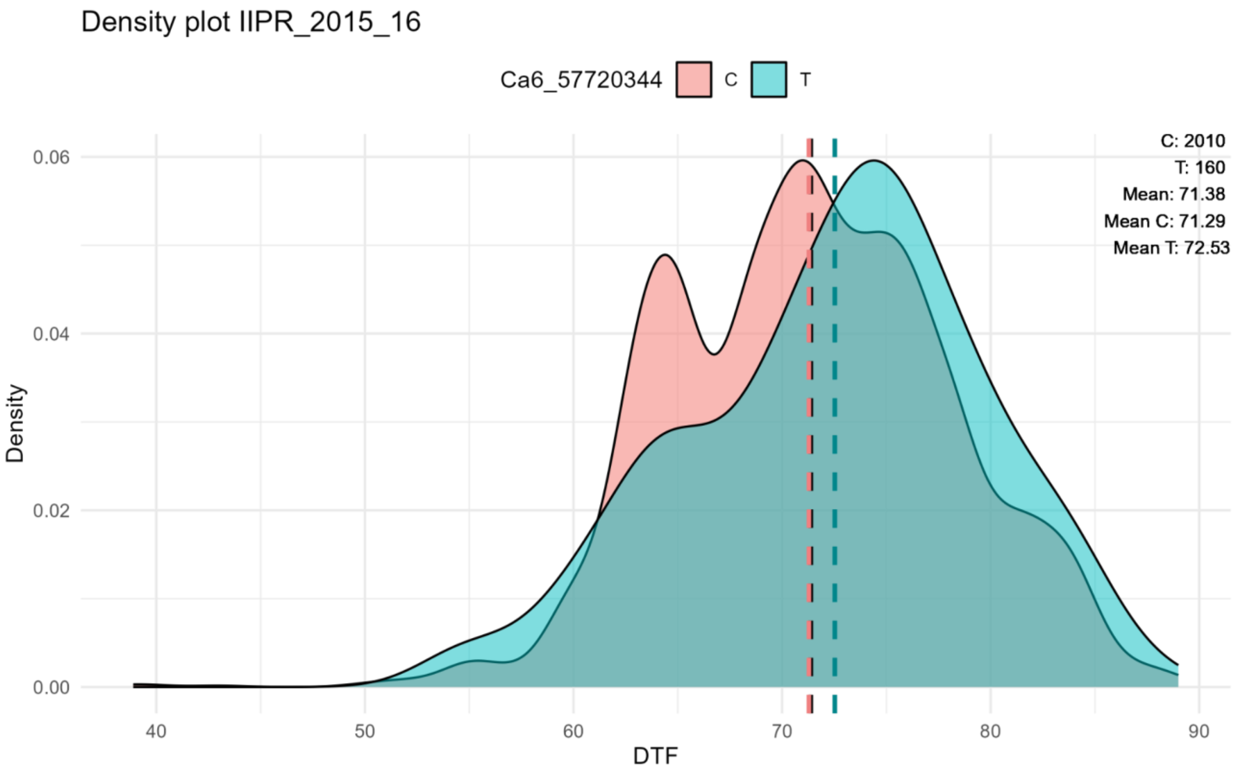

Density plot IIPR_2015_16
Ca6_57720344
C
T
C: 2010
T: 160
Mean: 71.38
Mean C: 71.29
Mean T: 72.53
0.06
0.04
0.02
0.00
Density
40
50
60
70
80
90
DTF

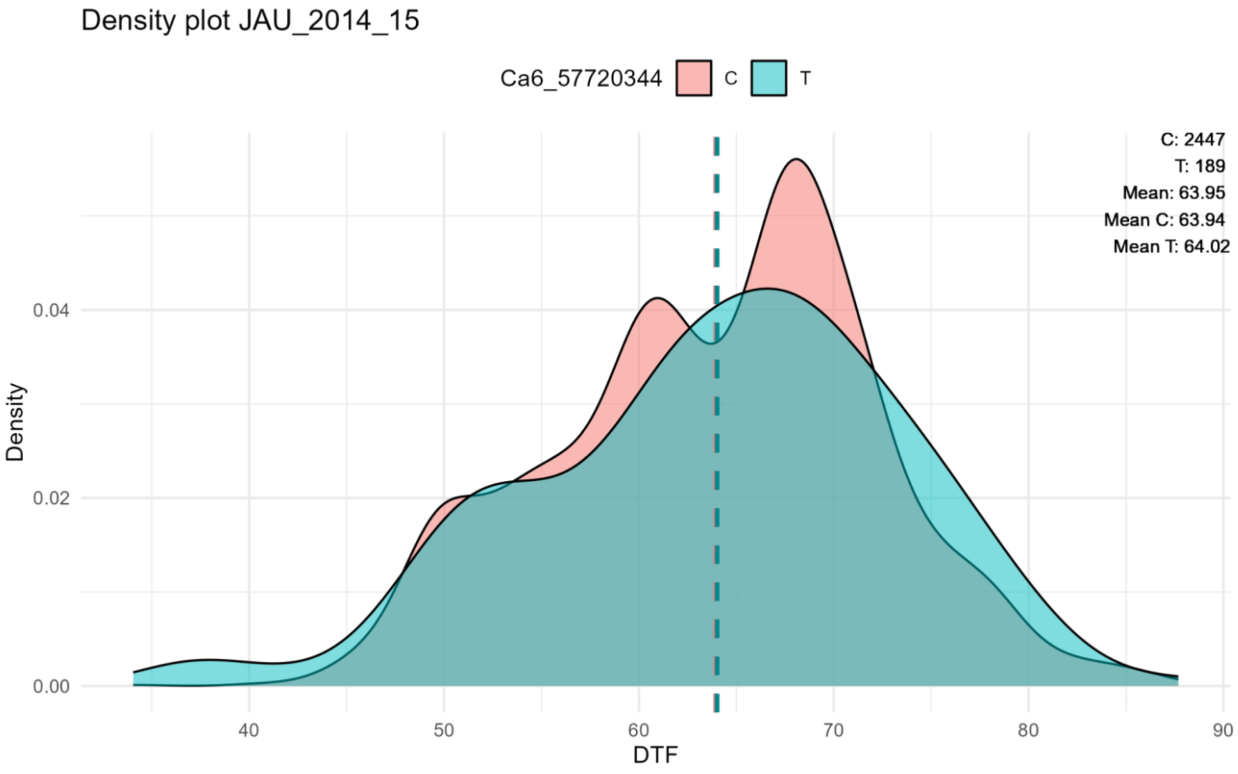

Density plot JAU_2014_15
Ca6_57720344
C
T
C: 2447
T: 189
Mean: 63.95
Mean C: 63.94
Mean T: 64.02
0.04
0.02
0.00
Density
40
50
60
70
80
90
DTF

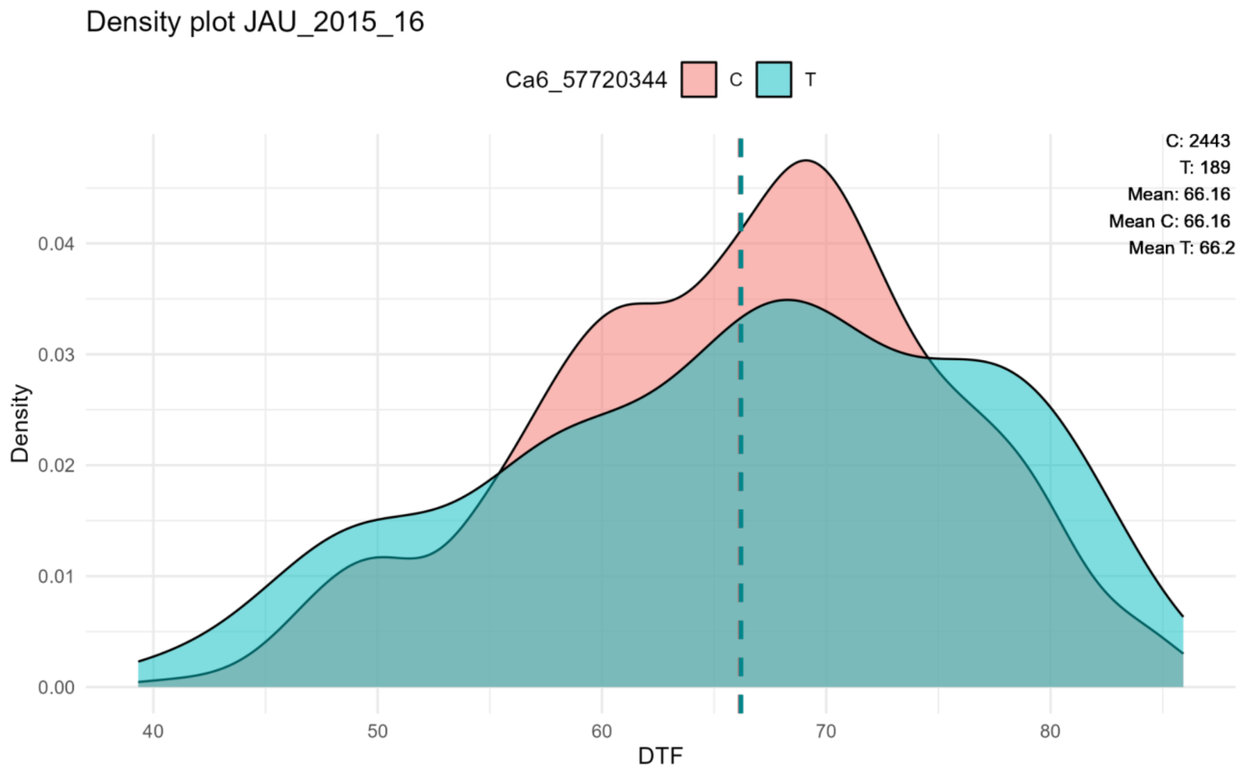
Density plot JAU_2015_16
Ca6_57720344
C
T
C: 2443
T: 189
Mean: 66.16
Mean C: 66.16
Mean T: 66.2
Density
0.00
0.01
0.02
0.03
0.04
40
50
60
70
80
DTF

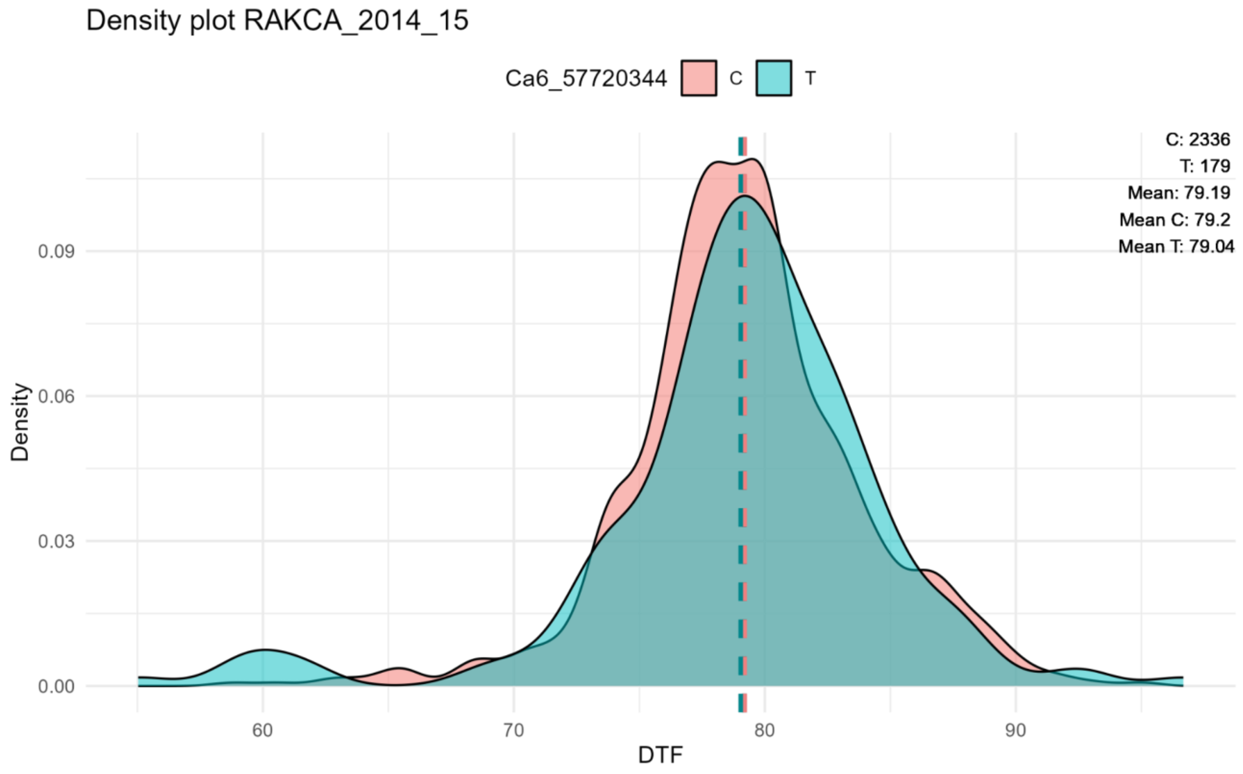
Density plot RAKCA_2014_15
Ca6_57720344
C
T
C: 2336
T: 179
Mean: 79.19
Mean C: 79.2
Mean T: 79.04
Density
0.00
0.03
0.06
0.09
60
70
80
90
DTF

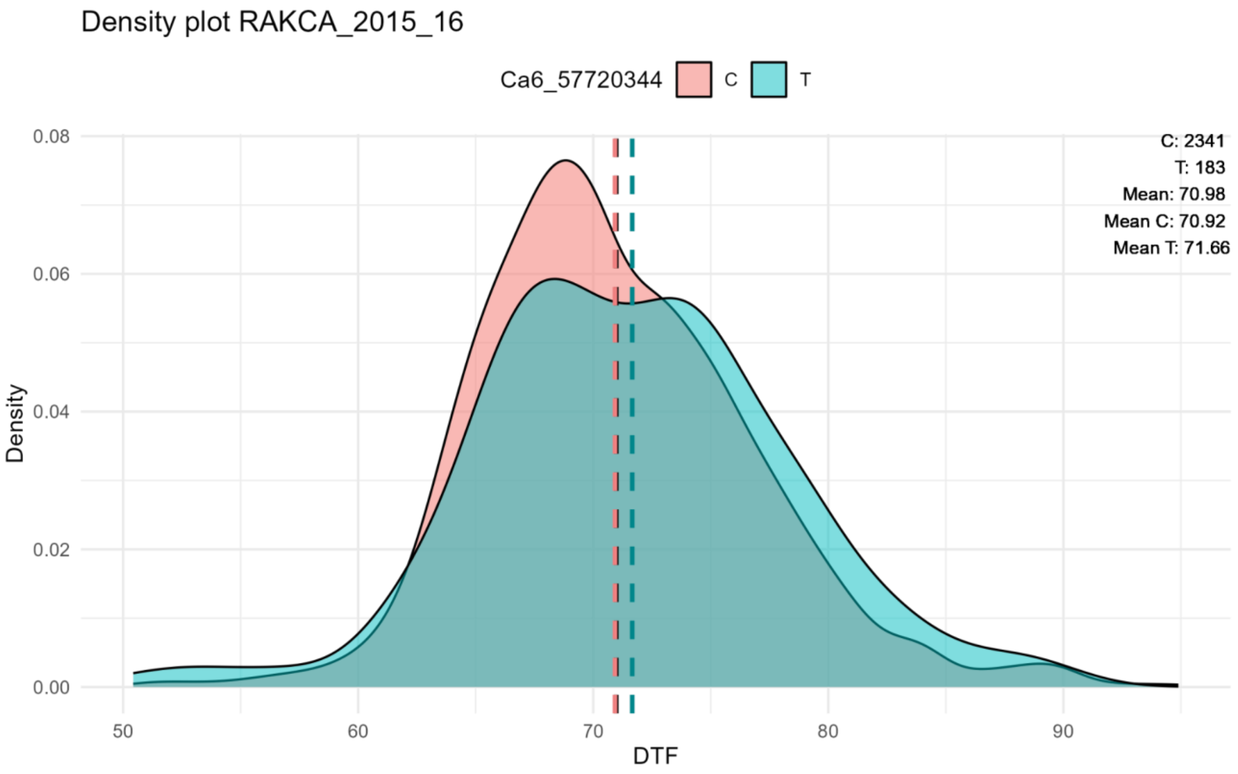
Density plot RAKCA_2015_16
Ca6_57720344
C
T
C: 2341
T: 183
Mean: 70.98
Mean C: 70.92
Mean T: 71.66
Density
0.08
0.06
0.04
0.02
0.00
50
60
70
80
90
DTF

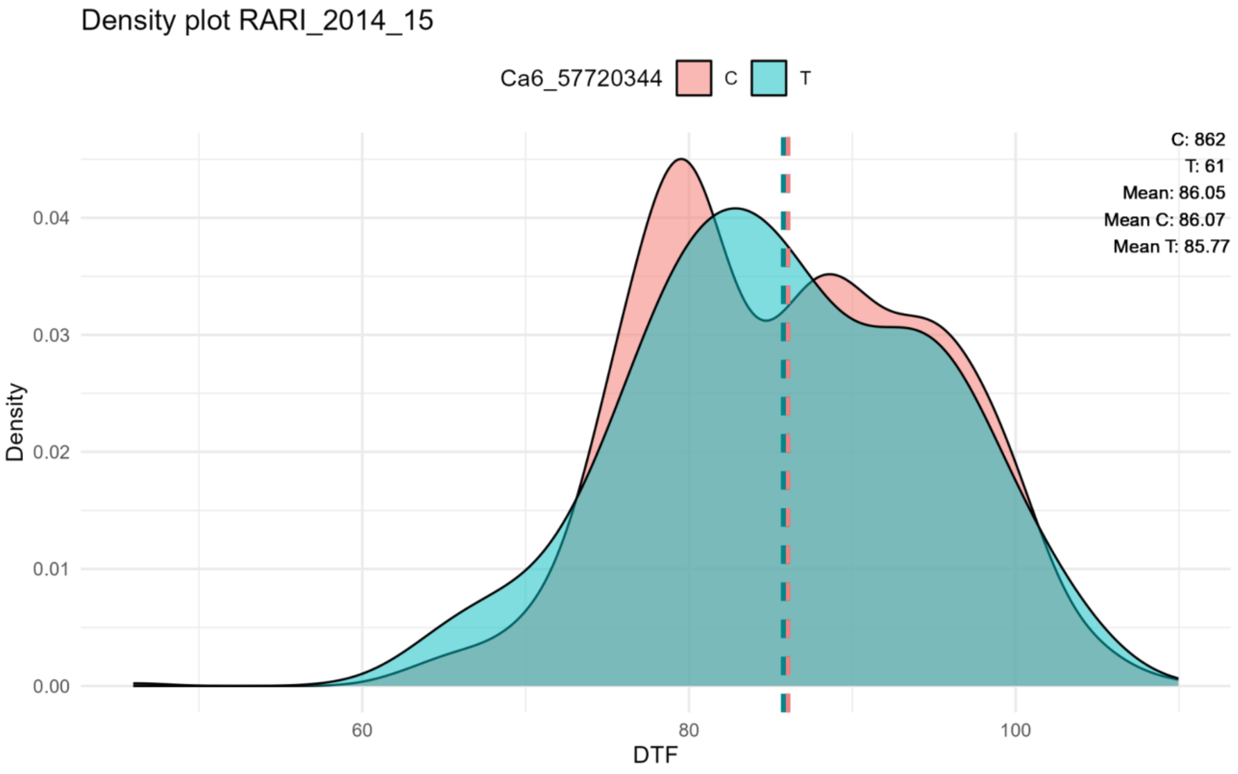
Density plot RARI_2014_15
Ca6_57720344
C
T
C: 862
T: 61
Mean: 86.05
Mean C: 86.07
Mean T: 85.77
Density
0.04
0.03
0.02
0.01
0.00
60
80
100
DTF

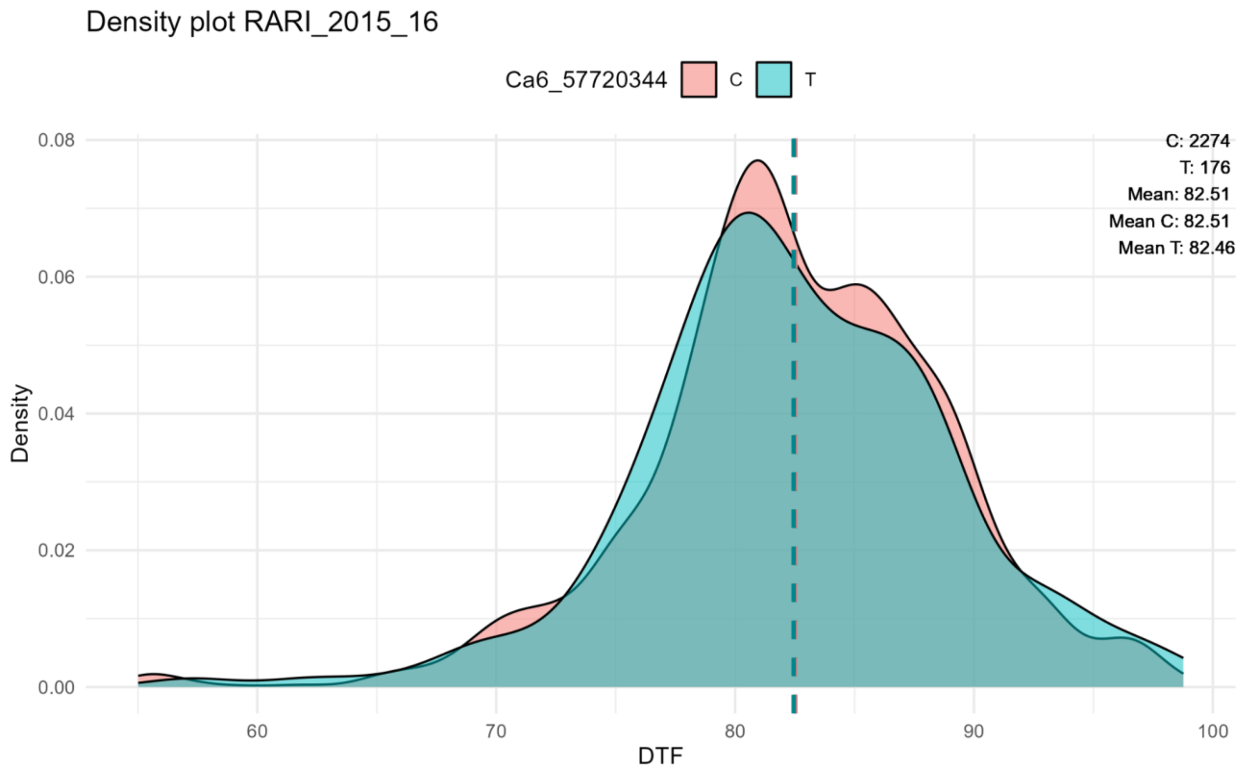

Additional file 10. Fig. S1 Density plot of cultivated chickpea accessions distribution based on days to flowering (DTF) according to (**a)** LOC101515142 Haplotype, (**b)** LOC101499101 SNP (Ca6: 57,549,449), and (**c)** LOC101507442 SNP (Ca6: 57,720,344). Vertical lines represent the global mean (black) and the means for each group (salmon and turquoise). The number of individuals taken into account for each location/year depending on the SNPs they present is indicated in the upper left corner of each of the plots.
