# Supplementary material for: Phenotypic and genetic characterization of a near-isogenic line pair: insights into flowering time in chickpea
Source: BMC Plant Biol. 2024 Jul 25;24:709. doi: 10.1186/s12870-024-05411-y (PMC11270784; doi:10.1186/s12870-024-05411-y)
Supplement: Supplementary file 11 — Additional file 11. Protein–protein interaction network using STRING chickpea database for (a) LOC101515142 and (b) LOC101499101. [file 12870_2024_5411_MOESM11_ESM.pdf]

a

LOC101515142

Mediator of RNA polymerase II transcription subunit 16-like

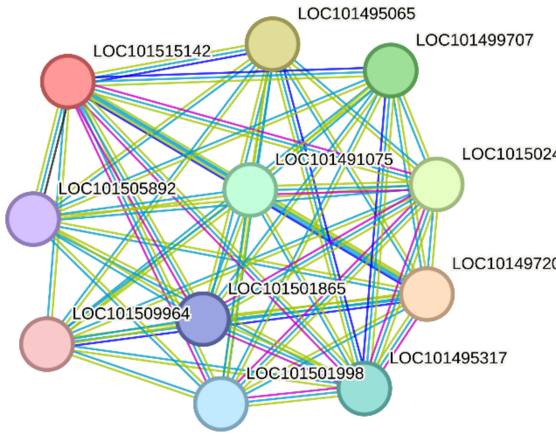

Edges:

Edges represent protein-protein associations

associations are meant to be specific and meaningful, i.e. proteins jointly contribute to a shared function; this does not necessarily mean they are physically binding to each other.

Predicted Interactions

- *gene neighborhood*
- *gene fusions*
- *gene co-occurrence*

Known Interactions

- *from curated databases*
- *experimentally determined*

Others

- *textmining*
- *co-expression*
- *protein homology*

Your Input:

LOC101515142 *Mediator of RNA polymerase II transcription subunit 16-like isoform X1. (1247 aa)*

Predicted Functional Partners:

Protein	Description	Neighborhood	Gene Fusion	Cooccurrence	Coexpression	Experiments	Databases	Textmining	[Homology]	Score
LOC101497203	*Mediator of RNA polymerase II transcription subunit 32.*			●			●	●		0.797
LOC101495065	*Mediator of RNA polymerase II transcription subunit 19a-like.*			●			●	●		0.708
LOC101502433	*Mediator of RNA polymerase II transcription subunit 14; Component of the Mediator complex, a coactivator involved in ...*					●	●	●		0.706
LOC101499707	*Probable mediator of RNA polymerase II transcription subunit 19b.*			●			●	●		0.691
LOC101491075	*Mediator of RNA polymerase II transcription subunit 18 isoform X1.*						●	●		0.670
LOC101495317	*Mediator of RNA polymerase II transcription subunit 27.*					●	●	●		0.640
LOC101501998	*Mediator of RNA polymerase II transcription subunit 8-like isoform X1.*					●	●	●		0.605
LOC101501865	*Mediator of RNA polymerase II transcription subunit 8-like isoform X1.*					●	●	●		0.605
LOC101505892	*Mediator of RNA polymerase II transcription subunit 13; Component of the Mediator complex, a coactivator involved in ...*				●		●	●		0.584
LOC101509964	*Mediator of RNA polymerase II transcription subunit 28.*						●	●		0.568

Your Current Organism:

Cicer arietinum

NCBI taxonomy Id: 3827

Other names: C. arietinum, Cicer arietinum L., chickpea, garbanzo

Network Stats

number of nodes:	11	expected number of edges:	10
number of edges:	52	PPI enrichment p-value:	< 1.0e-16
average node degree:	9.45		
avg. local clustering coefficient:	0.939		

Functional enrichments in your network

Biological Process (Gene Ontology)

GO-term	*description*	*count in network*	*strength*	*false discovery rate*
GO:0040034	Regulation of development, heterochronic	2 of 24	2.27	0.0056
GO:0009631	Cold acclimation	2 of 24	2.27	0.0056
GO:2000028	Regulation of photoperiodism, flowering	2 of 38	2.07	0.0128
GO:0009738	Abscisic acid-activated signaling pathway	4 of 106	1.93	2.52e-05
GO:0045944	Positive regulation of transcription by RNA polymerase II	5 of 207	1.74	1.76e-05
GO:0009909	Regulation of flower development	2 of 82	1.74	0.0473
GO:2000241	Regulation of reproductive process	4 of 200	1.65	0.00017
GO:0045893	Positive regulation of transcription, DNA-templated	6 of 402	1.53	1.76e-05
GO:0006357	Regulation of transcription by RNA polymerase II	8 of 557	1.51	4.60e-08
GO:0051239	Regulation of multicellular organismal process	3 of 253	1.43	0.0151
GO:0050793	Regulation of developmental process	5 of 438	1.41	9.44e-05
GO:0098542	Defense response to other organism	3 of 406	1.22	0.0497
GO:0006952	Defense response	4 of 812	1.04	0.0262
GO:0006355	Regulation of transcription, DNA-templated	9 of 2201	0.96	1.76e-05
GO:0006950	Response to stress	6 of 2436	0.74	0.0226
GO:0050789	Regulation of biological process	11 of 5261	0.67	1.76e-05

b

LOC101499101

B-box zinc finger protein 24

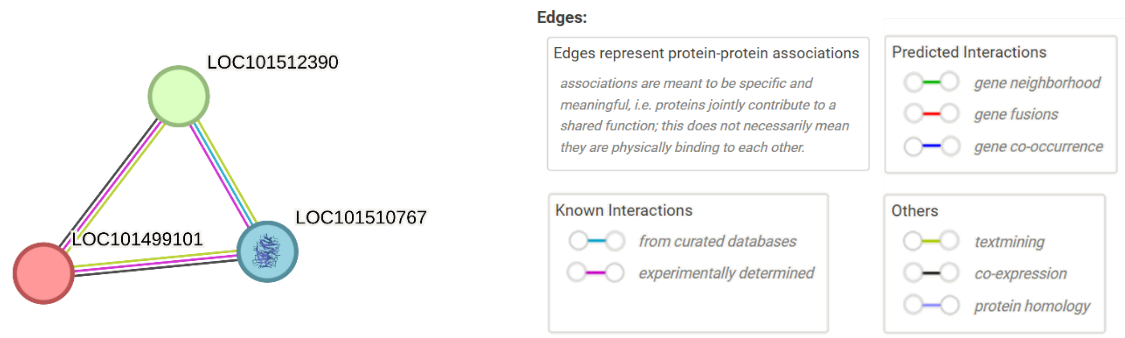

Your Input:

LOC101499101	*B-box zinc finger protein 24. (240 aa)*									

Predicted Functional Partners:

		Neighborhood	Gene Fusion	Cooccurrence	Coexpression	Experiments	Databases	Textmining	[Homology]	Score
LOC101512390	*Transcription factor HY5-like.*				●	●		●		0.440
LOC101510767	*E3 ubiquitin-protein ligase COP1.*				●	●		●		0.415

Your Current Organism:

Cicer arietinum

NCBI taxonomy Id: 3827

Other names: C. arietinum, Cicer arietinum L., chickpea, garbanzo

Network Stats

number of nodes:	3	expected number of edges:	2
number of edges:	3	PPI enrichment p-value:	0.212
average node degree:	2		
avg. local clustering coefficient:	1		

Functional enrichments in your network

Biological Process (Gene Ontology)

GO-term	description	count in network	strength	false discovery rate
GO:2000030	Regulation of response to red or far red light	2 of 17	2.99	0.0035
GO:0009585	Red, far-red light phototransduction	2 of 32	2.71	0.0051
GO:0009640	Photomorphogenesis	2 of 34	2.69	0.0051
GO:0010224	Response to UV-B	2 of 38	2.64	0.0051
GO:0009639	Response to red or far red light	3 of 106	2.37	0.00035

Additional file 11. Fig. S1 Protein-protein interaction network using STRING chickpea database for **(a)** LOC101515142 and **(b)** LOC101499101
